# Runx2 activates hepatic stellate cells to promote liver fibrosis via transcriptionally regulating *Itgav* expression

**DOI:** 10.1002/ctm2.1316

**Published:** 2023-07-05

**Authors:** Li Zhong, Jinqiu Zhao, Lu Huang, Yi Liu, Xiaoxiao Pang, Ke Zhan, Shan Li, Qian Xue, Xiaoli Pan, Liang Deng

**Affiliations:** ^1^ Department of Gastroenterology The First Affiliated Hospital of Chongqing Medical University Chongqing China; ^2^ Department of Gastroenterology and Hepatology The Second Affiliated Hospital of Chongqing Medical University Chongqing China; ^3^ Department of Infectious Diseases The First Affiliated Hospital of Chongqing Medical University Chongqing China; ^4^ Chongqing Key Laboratory of Child Infection and Immunity Chongqing China; ^5^ Department of Pediatric Research Institute Chongqing China; ^6^ Ministry of Education Key Laboratory of Child Development and Disorders Children's Hospital of Chongqing Medical University Chongqing China; ^7^ Chongqing Key Laboratory of Oral Diseases and Biomedical Sciences Chongqing China; ^8^ Chongqing Municipal Key Laboratory of Oral Biomedical Engineering of Higher Education Stomatological Hospital of Chongqing Medical University Chongqing China; ^9^ Department of Gastroenterology Union Hospital Tongji Medical College Huazhong University of Science and Technology Wuhan China

**Keywords:** TGF‐β1, Runx2, PKA, Itgav, HSC activation

## Abstract

**Backgrounds and aims:**

As a central event during liver fibrosis, hepatic stellate cells (HSC) have been thought to be a potential therapeutic target for liver fibrosis. Previous studies have shown that runt‐related transcription factor 2 (Runx2) is associated with the development of non‐alcoholic fatty liver disease, while its specific role in HSC activation and hepatic fibrosis remains elusive.

**Approach and results:**

In this study, we found that Runx2 expression was significantly upregulated in human liver fibrosis with different aetiologies. Runx2 expression was also gradually elevated in mouse liver during fibrosis, and Runx2 was mainly expressed in the activated HSC. Knockdown of Runx2 in HSC markedly alleviated CCl_4_‐induced, 3,5‐diethoxycarbonyl‐1,4‐dihydrocollidine‐induced or methionine‐choline deficient (MCD)‐induced liver fibrosis, while hepatic overexpression of Runx2 via HBAAV‐Runx2 or VA‐Lip‐Runx2 injection exacerbated CCl_4_‐induced liver fibrosis. In vitro analysis demonstrated that Runx2 promoted HSC activation and proliferation, whereas Runx2 knockdown in HSC suppressed these effects. RNA‐seq and Runx2 ChIP‐seq analysis demonstrated that Runx2 could promote integrin alpha‐V (*Itgav*) expression by binding to its promoter. Blockade of *Itgav* attenuated Runx2‐induced HSC activation and liver fibrosis. Additionally, we found that cytokines (TGF‐β1, PDGF, EGF) promote the expression and nuclear translocation of Runx2 through protein kinase A (PKA) in HSC.

**Conclusions:**

Runx2 is critical for HSC activation via transcriptionally regulating *Itgav* expression during liver fibrosis, and may be a promising therapeutic target for liver fibrosis.

## Graphical abstract

We identified Runx2 as a HSC‐specific protein during liver fibrosis, which is essential for the regulation of HSC activation. Targeted knockout of Runx2 expression in HSC or intrahepatic overexpression of Runx2 significantly inhibited or promoted liver fibrosis induced by various factors. Importantly, we demonstrated that PKA/Runx2/Itgav signaling is a probable mechanism for regulating HSC activation in liver fibrosis, and pharmacological blockade of Itgav attenuated Runx2‐induced HSC activation and liver fibrosis, which suggests a critical and yet unappreciated role of Runx2 in the development and progression of liver fibrosis, and may provide a potential new therapeutic target for liver fibrosis.

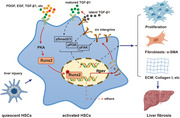

## 1 BACKGROUND

Liver fibrosis is a dynamic process characterised by abnormal accumulation of extracellular matrix (ECM), of which the major source is the activated hepatic stellate cells (HSC).^1^, ^2^ HSC reside in the Disse space and normally are maintained in a quiescent state.^3^ As liver injury starts, quiescent HSC are activated and transdifferentiated into highly proliferative, fibrogenic and contractile myofibroblasts upon the stimulation of profibrotic cytokines, such as transforming growth factor‐β1 (TGF‐β1), epidermal growth factors (EGF), platelet‐derived growth factor (PDGF).^4^, ^5^ Therefore, the activation of HSC is regarded as a central driver of liver fibrogenesis.^6^ Accumulated clinical and experimental evidences show that the elimination of activated HSC could lead to the resorption of the fibrous scar and resolve liver fibrosis.^2^, ^6^, ^7^ As a consequence, anti‐fibrotic therapies specifically targeting HSC activation become a promising strategy for liver fibrosis management.

The mechanism of HSC activation is a complex and well‐coordinated process that diverse pathways and mediators participate in, including autophagy, retinol and cholesterol metabolism, endoplasmic reticulum stress, oxidative stress, epigenetics, receptor‐mediated signals and transcriptional regulation.^6^, ^7^ We occasionally observed that the transcriptional protein, runt‐related transcription factor 2 (Runx2), was significantly increased during the progression of non‐alcoholic fatty liver (NAFLD) and predominantly expressed in activated HSC in the liver,^8^ which raises a question of whether Runx2 plays a critical role in the regulation of HSC activation. Runx2 is a DNA‐binding transcription factor that can regulate cell transformation by regulating multiple signalling pathways and transcriptional activation of a series of downstream molecules in various physiological and pathophysiological conditions, including bone formation, malignant tumours and vascular calcification.^9^, ^10^, ^11^, ^12^ Interestingly, Runx2 was shown to be associated with multiple‐organ fibrosis. For example, in type 2 diabetes, overexpression of Runx2 in vascular smooth muscle cells specifically increases matrix‐related target genes (collagen I) expression, thus promoting aortic fibrosis and stiffness.^13^ Yes‐associated protein‐mediated Runx2 activation is responsible for enhanced cardiac fibroblast proliferation in response to increased ECM stiffness^14^; on the other hand, Runx2 deficiency is shown to accelerate ureteral obstruction‐induced kidney fibrosis through activating the transforming growth factor‐β (TGF‐β) signalling pathway.^15^ Moreover, Runx2 expression is highly increased in fibrotic alveolar epithelial type II (AT II) cells, but not affected in fibroblasts during the process of pulmonary fibrosis, suggesting that the role of Runx2 in regulating fibrosis‐associated genes is opposite between AT II cells and fibroblasts.^16^ Taken together, Runx2 exhibits diverse functions in the fibrosis process of different organs and even exhibit a hallmark of cell‐specific expression in lung fibrosis. In the liver, a selective p38 mitogen‐activated protein kinase (MAPK) inhibitor is shown to ameliorate liver fibrosis through the downregulation of *Runx2* in a rat model,^17^ and Runx2 promotes epithelial–mesenchymal transition and vasculogenic mimicry in hepatocellular carcinoma,^18^ which indicates that Runx2 serves as a fibrogenic or tumorigenic factor in the liver. More importantly, upregulated Runx2 expression is found in the HSC during the development of NAFLD in a mouse model,^8^ and Runx2 increases tissue inhibitor of metalloproteinase‐1 (TIMP‐1) expression by binding to its promoter in the activated HSC of rats.^19^ However, the specific role and underlying molecular mechanism of Runx2 during HSC activation and liver fibrosis remain elusive. In this study, we identified that Runx2 was specifically expressed in HSC and is essential for HSC activation. Knockdown or overexpression of Runx2 expression in HSC significantly inhibited or promoted liver fibrosis induced by various factors. Additionally, we demonstrated the mechanism of Runx2 nuclear translocation and regulation of HSC activation. Our data revealed a critical and yet unappreciated role of Runx2 in liver fibrosis and may provide a potential new therapeutic target for liver fibrosis.

## 2 MATERIALS AND METHODS

### 2.1 Human liver samples

Human liver non‐fibrosis or cirrhosis samples were obtained from individuals who had undergone liver surgery or liver transplantation. It contained 10 cases of liver cirrhosis and 10 non‐fibrosis liver tissues. Tissue specimens were dissected and flash‐frozen in liquid nitrogen until the isolation of total RNA or protein. Written informed consent was obtained from each patient. Human sample collection was consistent with the Declaration of Helsinki, and was approved by the ethics committee of the First Affiliated Hospital of Chongqing Medical University. Clinical characteristics (age, sex, cirrhosis grade and disease type) of each patient are provided in Table S1.

### 2.2 Transgenic mouse

The Animal Experimental Center of Chongqing Medical University provided C57BL/6 male mice that were 4−6 weeks old. *Runx2*‐floxed (*Runx2^f/f^*) mice, in which the exon 4 of the Runx2 allele was flanked by loxP sites, were purchased from Viewsolid Biotech (Beijing, China). *GFAP‐Cre* and *Alb‐Cre* mice were purchased from Viewsolid Biotech (Beijing, China). Platelet‐derived growth factor receptor β (*PDGFRβ*)*‐Cre* mice were provided by Kunfu Ouyang from Drug Discovery Center, State Key Laboratory of Chemical Oncogenomics, School of Chemical Biology and Biotechnology, Peking University Shenzhen Graduate School. HSC‐specific Runx2 knockout (*Runx2^△/△HSC^*) mice on the C57BL/6 background were generated by intercrossing *Runx2^f/f^* mice with the *PDGFRβ‐Cre* or *GFAP‐Cre* mice. HC‐specific Runx2 knockout (*Runx2^△/△HC^*) mice were generated by intercrossing *Runx2^f/f^* mice with the *Alb‐Cre* mice. To overexpress Runx2 in the liver, an Adeno‐associated virus vector (Hanbio, China) encoding the cDNA of murine Runx2 (*HBAAV‐Runx2*) or empty control vector (*HBAAV‐control*) was injected via the portal vein (5 × 10^12^ virus particles/mouse). Frozen sections of liver tissues were observed by fluorescence microscopy to determine the transfection efficiency. Genotyping primer sequences are provided in Tables S2 and S3.

All mice received humane care and were bred under specific pathogen‐free conditions. Animal studies were approved by the Animal Care and Use Committee at Chongqing Medical University. The animal experimental procedures were performed according to the National Institutes of Health Guidelines for the Use of Experimental Animals and approved by the Ethics Committee and the Medicine Animal Care Committee of Chongqing Medical University. A detailed procedure is found in the Supporting Materials and Methods.

More detailed information regarding the experimental procedures is included in the Supporting Information.

## 3 RESULTS

### 3.1 Runx2 expression is progressively increased during the progression of liver fibrosis

**FIGURE 1:**
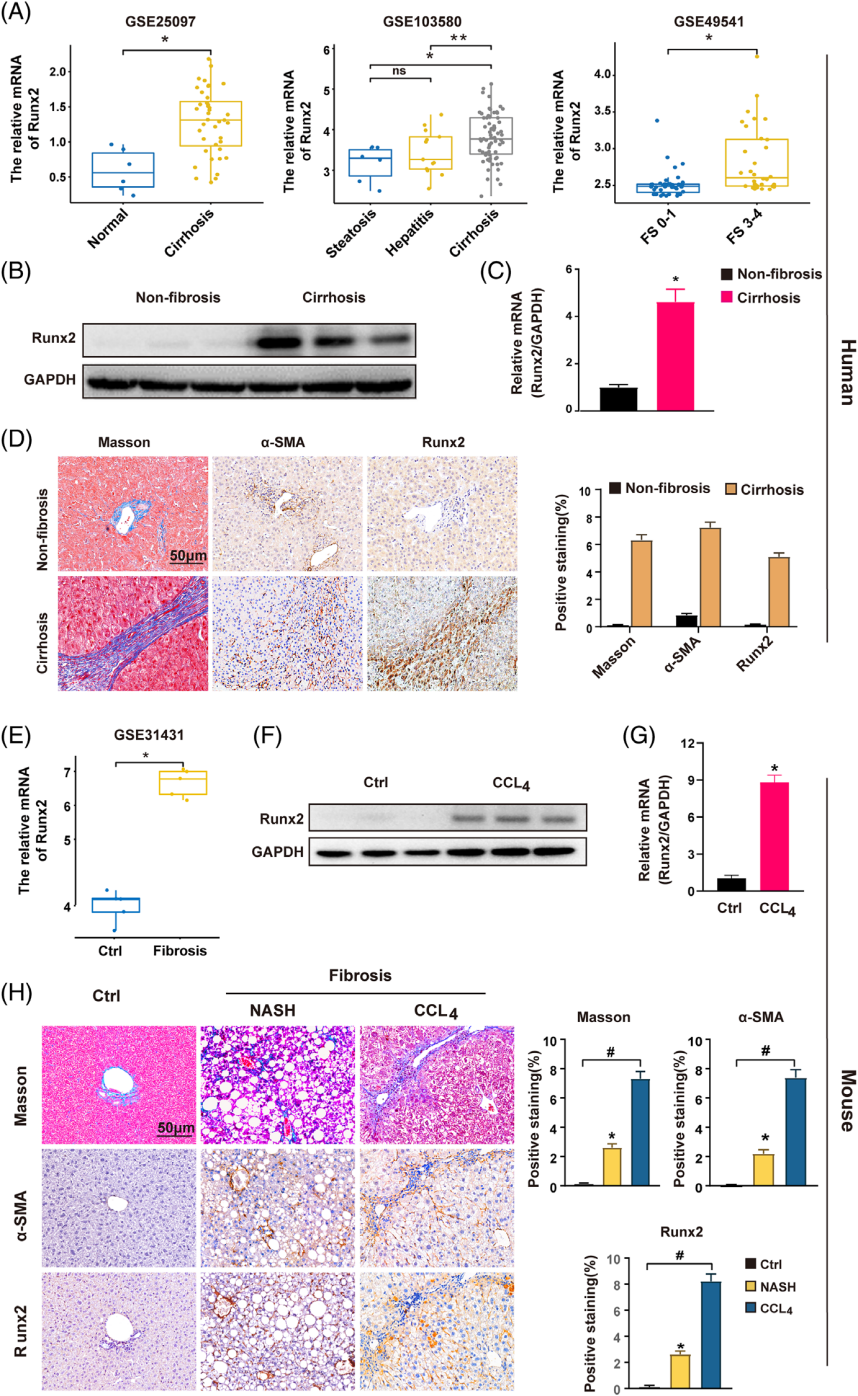
The expression of Runx2 was gradually increased during the progression of liver fibrosis. (A) The mRNA expression of Runx2 in human liver cirrhosis tissues in GSE25097 cohorts (left), human liver tissues with alcoholic hepatitis or cirrhosis in GSE103580 cohorts (middle), and human liver fibrosis tissues at different fibrosis stage (FS) in GSE49541cohorts (right) compared to control samples. (B and C) The protein and mRNA levels of Runx2 in human non‐fibrosis or cirrhosis liver samples were detected by Western blot assay and qRT‐PCR (*n* = 3). (D) Human hepatic tissues were collected from individuals with non‐fibrosis or cirrhosis. Representative histology of Masson and IHC staining of α‐SMA and Runx2 in liver tissues of each group are presented. Quantification of positive staining areas was measured by ImageJ software. Scale bar: 50 μm (*n* = 10). (E) The mRNA expression of Runx2 in mouse liver fibrotic tissues in GSE31431cohorts compared to control samples. (F and G) Mice liver fibrosis was induced by intraperitoneal (IP) injection of CCl_4_ (5 μL/g bodyweight) for 4 weeks. The protein and mRNA levels of Runx2 in liver tissues were examined by Western blot assay and qRT‐PCR (*n* = 3). (H) Mice were fed a high‐fat diet for 12 months to induce NASH‐related fibrosis. And, mice liver fibrosis was induced by IP injection of CCl_4_ for 4 weeks. Representative histology of Masson and IHC staining of α‐SMA and Runx2 in liver tissues of each group are shown. Quantification of positive staining areas was measured by ImageJ software. Scale bar: 50 μm (*n* = 6). Data are mean ± SEM; ^*^p, ^**^p < .05 versus controls; *^#^p* < .01; ns, non‐significant.

To investigate the role of Runx2 in liver fibrosis, we first explored Runx2 expression in the GEO database. The production of Runx2 was considerably higher in patients with liver cirrhosis caused by different aetiology, such as alcoholic liver disease, viral hepatitis and NAFLD (Figure 1A). We also found that the level of Runx2 was higher in the individuals with advanced fibrosis stage, whereas lower in patients at early fibrosis stage, indicating that Runx2 expression was increased during progression of liver fibrosis (Figure S1). Then, human liver tissues were collected from individuals with non‐fibrosis or cirrhosis, and data consistently revealed that protein and mRNA levels of Runx2 were significantly upregulated in cirrhotic liver tissues (Figure 1B,C). Histologically, Masson's trichrome staining showed a severe collagen deposition in cirrhotic tissues, accompanied by markedly increasing Runx2 and α‐SMA (a marker for activated HSC) expression. Runx2 was positively stained at fibrous cords (Figure 1D). Similar result with upregulated Runx2 expression was observed in fibrotic liver tissues both in the GEO database and mice fibrosis model caused by CCl_4_ (Figure 1E–H). More importantly, we found that Runx2 and α‐SMA were both markedly increased in non‐alcoholic steatohepatitis (NASH)‐related liver fibrosis induced by high‐fat diet treatment (Figure 1H).

**FIGURE 2:**
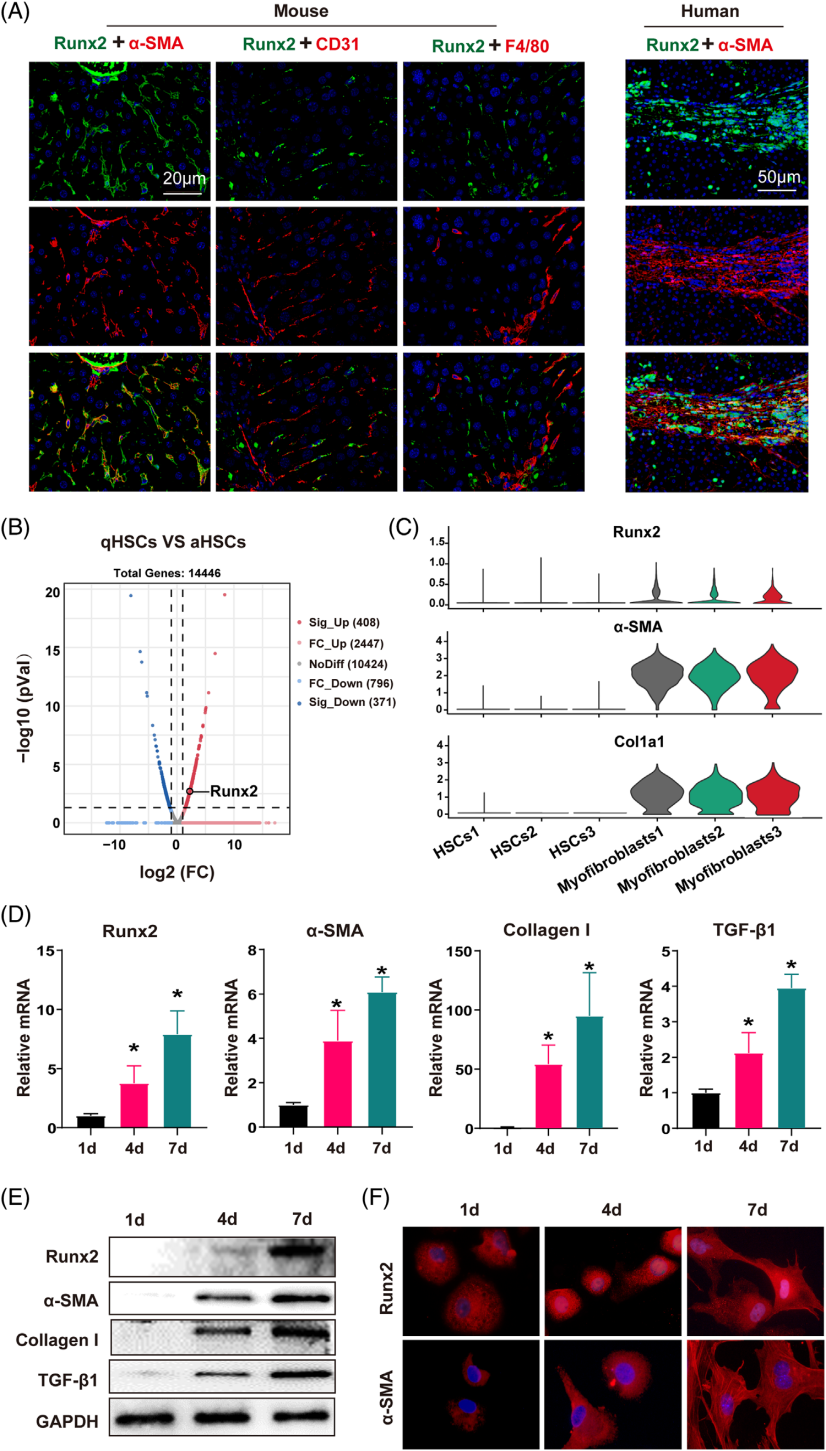
Runx2 is specifically located in activated HSC in vivo, and increased in a time‐dependent manner during HSC activation in vitro. (A) Immunofluorescence micrographs of liver sections from CCl_4_‐treated mice and cirrhotic patients, staining for activated HSC (α‐SMA, red), endothelium (CD31, red) and Kupffer cells (F4/80, red), with Runx2 in green. Scale bars: 20 μm for mice; 50 μm for humans (*n* = 3). (B) Primary HSC were isolated from the mice treated with olive or CCl_4_ for 4 weeks, then the quiescent HSC (qHSC, olive) and activated HSC (aHSC, CCl_4_) were examined by RNA‐sequencing analysis, and Volcano Plot analysis is presented. (C) Violin plots showing the relative expression of selected marker genes (Runx2, α‐SMA and Col1a1) for each cluster; average of 1000 cells per condition. (D and E) Primary HSC were isolated from normal mouse liver and cultured for the indicated time intervals (first, fourth and seventh days). The protein and mRNA expressions of Runx2, α‐SMA, collagen I and TGF‐β1 were examined by Western blot and qRT‐PCR (*n* = 5). (F) Immunofluorescence staining of Runx2 and α‐SMA in primary HSC cultured for the indicated time intervals. High‐magnification images are shown (*n* = 3). Data are mean ± SEM; ^*^p < .05 versus controls.

To further investigate the tendency of Runx2 expression during liver fibrosis, mice were treated with CCl_4_ for 1 week and 6 weeks to induce liver injury and fibrosis, and Runx2 expression was monitored. We found that collagen deposition progressively increased as liver injury persisted, and the expression of α‐SMA and Runx2 was mildly upregulated in the injured liver, but markedly provoked in fibrotic liver, which was consistent with the analysis from the GEO database (Figure 2S). Interestingly, the histological staining consistently indicated that the positive staining of Runx2 was predominantly distributed in the fibrous cords and septa in liver tissues, coinciding with the distribution of α‐SMA positive cells (Figure 1D,H). Collectively, these findings indicated that Runx2 expression was gradually increased during the liver fibrosis process, and presented potentially cell‐specific expression in liver tissues.

### 3.2 Runx2 specifically located in activated HSC in vivo, and increased in a time‐dependent manner during HSC activation in vitro

Since Runx2 was predominantly expressed in liver interstitial cells, coinciding with α‐SMA expression; we co‐stained Runx2 with α‐SMA (activated HSC marker), CD31 (endothelial cells marker) or F4/80 (Kupffer cells marker) in the fibrotic liver of mice to assess site‐specificity of Runx2 in the liver. As predicted, the co‐existence phenomenon was only observed between Runx2 and α‐SMA, which was further confirmed in human cirrhotic liver tissues (Figure 2A). Additionally, by comparing Runx2 expression between quiescent HSC and activated HSC isolated from mice treated with olive or CCl_4_ for 4 weeks, we found that Runx2 expression was significantly increased in the activated HSC (Figure 2B). Notably, single‐cell RNA sequencing analysis revealed that Runx2 was highly expressed and distributed in myofibroblasts (well‐differentiated HSC) compared to the primary HSC cultured in the early stage consistent with myofibroblasts markers (α‐SMA and collagen type I alpha 1 chain [Col1a1]), which further supports that Runx2 mainly existed in the activated HSC (Figure 2C and Figure S3). Most importantly, mRNA and protein expression of Runx2 were upregulated in a time‐dependent manner during HSC activation, which is associated with the expression of fibrotic genes including α‐SMA, collagen I and TGF‐β1 (Figure 2D,E). Meanwhile, we observed that Runx2 showed excessive nuclear translocation accompanied by HSC activation in vitro, implying that Runx2 started its transcriptional function concomitantly (Figure 2F). Taken together, these results revealed that Runx2 is specifically expressed in the activated HSC and may play a role in regulating HSC activation.

### 3.3 HSC‐specific knockdown of Runx2 alleviates CCl_4_‐induced, DDC‐induced or MCD‐induced liver fibrosis in mice

**FIGURE 3:**
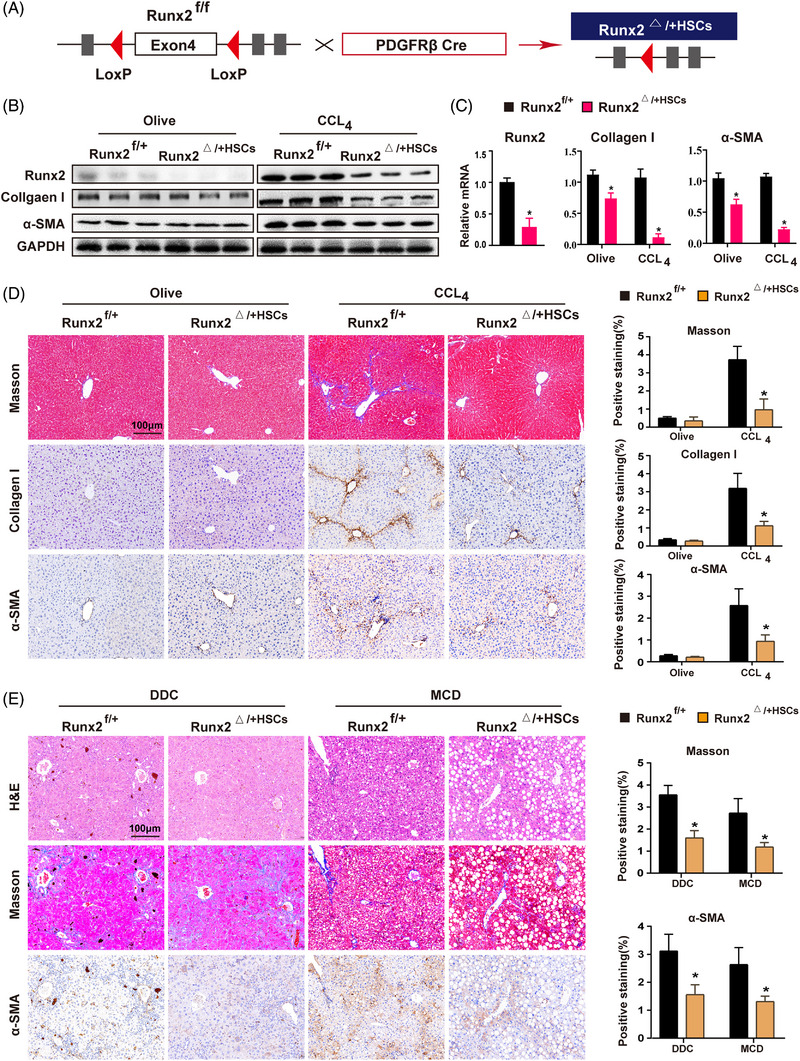
HSC‐specific knockdown of Runx2 alleviates CCl_4_‐induced, DDC‐induced or MCD‐induced liver fibrosis in mice. (A) Schematic showing the strategy for generating HSC‐specific knockdown of Runx2 mice (*Runx2^△+HSC^*). (B and C) Western blot and qRT‐PCR showed the protein and mRNA levels of Runx2, collagen I and α‐SMA in *Runx2^f+^* and *Runx2^△+HSC^* mice treated with olive or CCl_4_ for 4 weeks (*n* = 3). (D) Representative photomicrographs of Masson and IHC staining of collagen I and a‐SMA in liver tissues from *Runx2^f+^* and *Runx2^△+HSC^* mice treated with olive or CCl_4_. Positive staining areas were measured by ImageJ software. Scale bars: 100 μm (*n* = 5). (E) *Runx2^f+^* and *Runx2^△+HSC^* mice were fed with .1% DDC for 4 weeks to induce cholestasis, or with MCD for 8 weeks to induce NASH. Representative photomicrographs of H&E, Masson and IHC staining of a‐SMA are shown. Positive staining areas were measured by ImageJ software. Scale bars: 100 μm (*n* = 3). Data are mean ± SEM; *p < .05 versus controls.

To determine the role of Runx2 in the process of liver fibrosis in vivo, we first utilised lenti‐shRNA to knock down Runx2 in mice treated with CCl_4_, and the data indicated that Runx2 knockdown alleviated CCl_4_‐induced liver fibrosis (Figure S4). Next, HSC‐specific Runx2‐ablated mice were generated by using *PDGFRβ‐Cre* mice, which is recognised as an HSC‐specific gene.^20^, ^21^, ^22^, ^23^ However, the homozygotes (*Runx2^ff^*; *PDGFRβ‐Cre*) died immediately after born, so the heterozygotes of Runx2 half deletion mice (*Runx2^f+^*; *PDGFRβ‐Cre* or *Runx2* ^***△+HSC***^) were used for further in vivo investigations (Figure 3A and Figure S5A). The results indicated that Runx2 knockdown decreased mRNA and protein expression of the profibrotic genes, including TGF‐β1, collagen I and α‐SMA in fibrotic groups induced by CCl_4_ treatment. Although their mRNA expression was mildly decreased in *Runx2* ^***△+HSC***^ mice treated with olive, the protein levels of these genes showed no significant changes (Figure 3B,C). Additionally, Runx2 knockdown significantly alleviated CCl_4_‐induced liver fibrosis histologically with decreased collagen I deposition and HSC activation (Figure 3D). Furthermore, 3,5‐diethoxycarbonyl‐1,4‐dihydrocollidine (DDC)‐induced or methionine‐choline‐deficient (MCD)‐induced liver fibrosis mice models were established to evaluate the role of Runx2 in liver fibrosis caused by other aetiologies.^24^, ^25^ The H&E staining verified that DDC or MCD successfully induced cholestasis or NASH in mice, and the level of fibrosis and HSC activation was significantly lower in Runx2‐deficient mice compared to the controls (Figure 3E). Similarly, Runx2 deficiency reduced mRNA and protein expression of collagen I and α‐SMA in DDC‐ and MCD‐induced fibrotic liver tissues (Figure S6). Moreover, as hepatocytes (HCs) damage is an integral part of liver fibrosis, we generated HC‐specific Runx2 knockout mice (Runx2^△/△HCs^) by crossing *Runx2^ff^* mice with *Alb‐Cre* mice to verify whether Runx2 ablation in HCs affects the progress of liver fibrosis. Expectedly, HC‐specific deletion of Runx2 did not affect CCl_4_‐induced liver fibrosis (Figure S7). Collectively, these findings suggested that Runx2 knockdown in HSC alleviates CCl_4_‐induced, DDC‐induced and MCD‐induced hepatic fibrosis.

### 3.4 Overexpression of Runx2 exacerbates CCl_4_‐induced liver fibrosis

**FIGURE 4:**
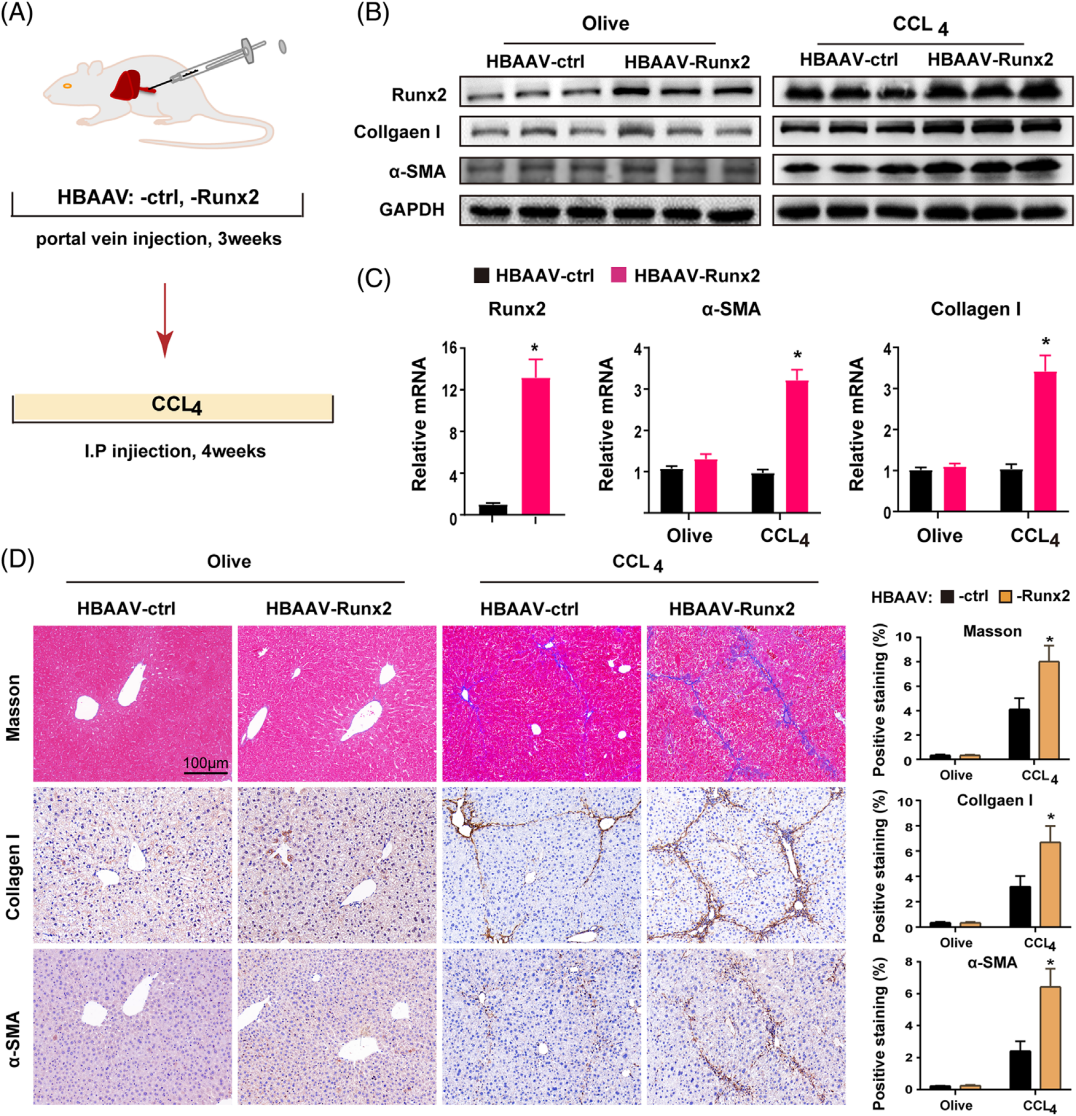
Runx2 overexpression exacerbated CCl_4_‐induced liver fibrosis. (A) The illustration of experimental design for overexpression of Runx2 in liver fibrosis. Mice were subjected to olive or CCl_4_ for 4 weeks after being injected by HBAAV‐ctrl or HBAAV‐Runx2 (100 μL per mouse, 1 × 10^12^ V g/mL, portal vein) for 3 weeks. (B and C) The protein and mRNA expressions of Runx2, collagen I and α‐SMA were detected by Western blot and qRT‐PCR (*n* = 3). (D) Representative images of Masson and IHC staining of collagen I and a‐SMA in *HBAAV‐ctrl* or *HBAAV‐Runx2* mice treated with olive or CCl_4_. Quantification of positive staining areas was measured by ImageJ software. Scale bars: 100 μm (*n* = 5). Data are mean ± SEM; *p < .05 versus controls.

To further confirm the role of Runx2 in liver fibrosis, Runx2 overexpression mice were generated by injecting HBAAV‐Runx2 (1 × 10^12^ V g/mL, portal vein) for 3 weeks, and then the mice were subjected to olive or CCl_4_ for 4 weeks (Figure 4A). The transfection efficiency was determined by fluorescence observation of frozen sections of mice after sacrifice (Figure S8A). Subsequently, primary HSC and HC were isolated and the mRNA expression of Runx2 was detected to clarify the transduced cell populations and delivery efficiency in the liver. The results showed that the majority of the HBAAV‐Runx2 were transfected into HC compared to HSC (Figure S8B). However, as Runx2 did not influence HC as described previously, to some extent we successfully generated Runx2 overexpression mice in HSC. Following CCl_4_ treatment, the mRNA and protein expressions of Runx2, collagen I and α‐SMA were significantly increased in *HBAAV‐Runx2* mice compared to controls (Figure 4B,C). Histologically, the liver structures in the *HBAAV‐Runx2* group displayed extensive structural disorganisation and significant gaps between adjacent vascular structures compared with the control group (Figure 4D). Positive staining of collagen I and α‐SMA cells was also increased in the *HBAAV‐Runx2* mice, indicating that fibrosis was severe and a greater number of HSC were activated (Figure 4D). Furthermore, we found that Runx2 overexpression triggered the proliferation of glial fibrillary acidic protein (GFAP)‐positive cells (quiescent HSC) in mice treated with olive oil, but did not affect the collagen deposition, HSC activation, mRNA and protein expression of collagen I and α‐SMA (Figure 4D and Figure S9), indicating that Runx2 overexpression in HSC could not trigger spontaneous fibrosis or HSC activation. Additionally, we found that Runx2‐specific overexpression in HSC enhanced CCl_4_‐induced liver fibrosis in mice by the injection of vitamin A‐coupled liposomes carrying Runx2 plasmid (VA‐Lip‐Runx2) or VA‐Lip‐Ctrl as well (Figure S10). Together, the above results revealed that overexpression of Runx2 exacerbates CCl_4_‐induced liver fibrosis.

### 3.5 Runx2 regulates the activation of HSC in vitro

**FIGURE 5:**
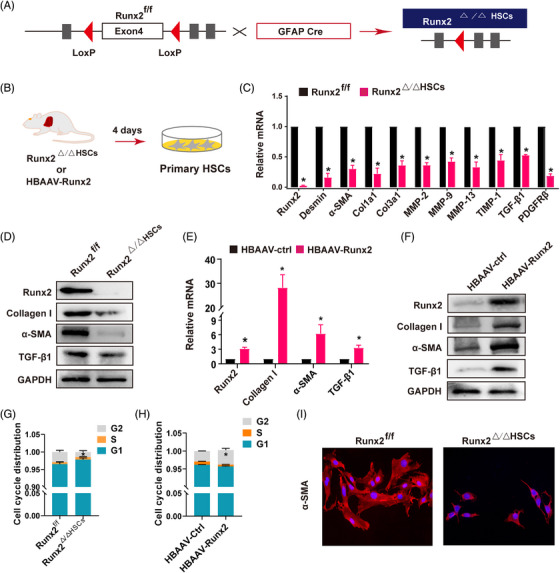
Runx2 regulated the activation of HSC in vitro. (A) Schematic showing the strategy for generating HSC‐specific deletion of Runx2 mice (*Runx2^△/△HSC^*). (B) Primary HSC were isolated from *Runx2^△/△HSC^* or *HBAAV‐Runx2* mice and cultured for 4 days under 3% FBS condition. (C) Fibrogenic‐related genes were measured by qRT‐PCR assays in primary HSC isolation from *Runx2^f/f^* or *Runx2^△/△HSC^* mice. (D) Immunoblotting assays of Runx2, α‐SMA, collagen I and TGF‐β1 in primary HSC isolating from *Runx2^f/f^* or *Runx2^△/△HSC^* mice. (E and F) The mRNA and protein expressions of Runx2, α‐SMA, collagen I and TGF‐β1 were measured by qRT‐PCR and Western blot assay in primary HSC isolated from *HBAAV‐ctrl* or *HBAAV‐Runx2* mice. (G and H) The percentage of exponentially growing in each cell cycle phase of primary HSC was measured by flow cytometry analysis. (I) The activation characteristic was determined by immunofluorescence staining of α‐SMA in primary HSC isolated from *Runx2^f/f^* or *Runx2^△/△HSC^* mice. Data are mean ± SEM; *n* = 3; ^*^p < .05 versus controls.

Since *Runx2^f/f^* mice crossed with *PDGFRβ‐Cre* mice only bred out Runx2 half‐ablated mice (*Runx2^△/+HSC^*); we utilised *GFAP‐Cre* (another marker for HSC in the liver) mice to cross with *Runx2^f/f^* mice and successfully got Runx2 deletion mice (*Runx2^△/△HSC^*) (Figure 5A and Figure S5B). Given the fact that GFAP‐Cre reporters also labelled bile ducts and cytokeratin 19‐expressing cholangiocytes,^23^ *Runx2^△/△HSC^* mice were mainly used for in vitro experiments in our study. Undoubtedly, we found that the fibrosis was acutely alleviated in *Runx2^△/△HSC^* mice treated with CCl_4_ for 4 weeks (Figure S11). To further evaluate the role of Runx2 in HSC activation, primary HSC were isolated from *Runx2* ***^△^^/^^△^^HSC^*** mice or *HBAAV‐Runx2* mice, and cultured for 4 days (Figure 5B). We found that the mRNA expression of HSC marker genes and fibrotic genes such as desmin, α‐SMA, Col1a1, collagen type III alpha 1 chain (Col3a1), matrix metalloproteinase 2 (MMP2), MMP9, MMP13, TIMP‐1, TGF‐β1 and PDGFRβ were reduced in HSC of *Runx2^△/△HSC^* mice (Figure 5C), as well as the decreased protein expression of α‐SMA, collagen I and TGF‐β1 was observed in the Runx2‐deleted HSC (Figure 5D), while the mRNA and protein expression of fibrotic genes such as α‐SMA, collagen I and TGF‐β1 was increased in HSC of Runx2 overexpression mice (Figure 5E,F). Besides, Runx2 deletion suppressed α‐SMA expression, and maintained a more quiescent phenotype of HSC in vitro (Figure 5I). We further used small interference to knockdown Runx2 in primary HSC, LX2 (human HSC cell line) or mHSC (mouse HSC cell line), and the results revealed that Runx2 deletion decreased the mRNA expression of α‐SMA in all of the cells, while Runx2 overexpression upregulated the mRNA expression of α‐SMA in the mHSC cell line (Figure S12A,B). Additionally, the flow cytometry analysis showed that Runx2 ablation significantly reduced the G2 phase of HSC, indicating that HSC proliferation was inhibited, but overexpression of Runx2 consistently deceased S phase of HSC and increased G2 phase of HSC, and significantly promoted the HSC growth in vitro (Figure 5G and Figure S12C,D), indicating that Runx2 was a positive regulator for HSC proliferation. Taken together, these findings suggested that Runx2 was an essential transcriptional factor for HSC activation and proliferation.

### 3.6 Itgav is a direct downstream target of Runx2 in liver fibrosis

**FIGURE 6:**
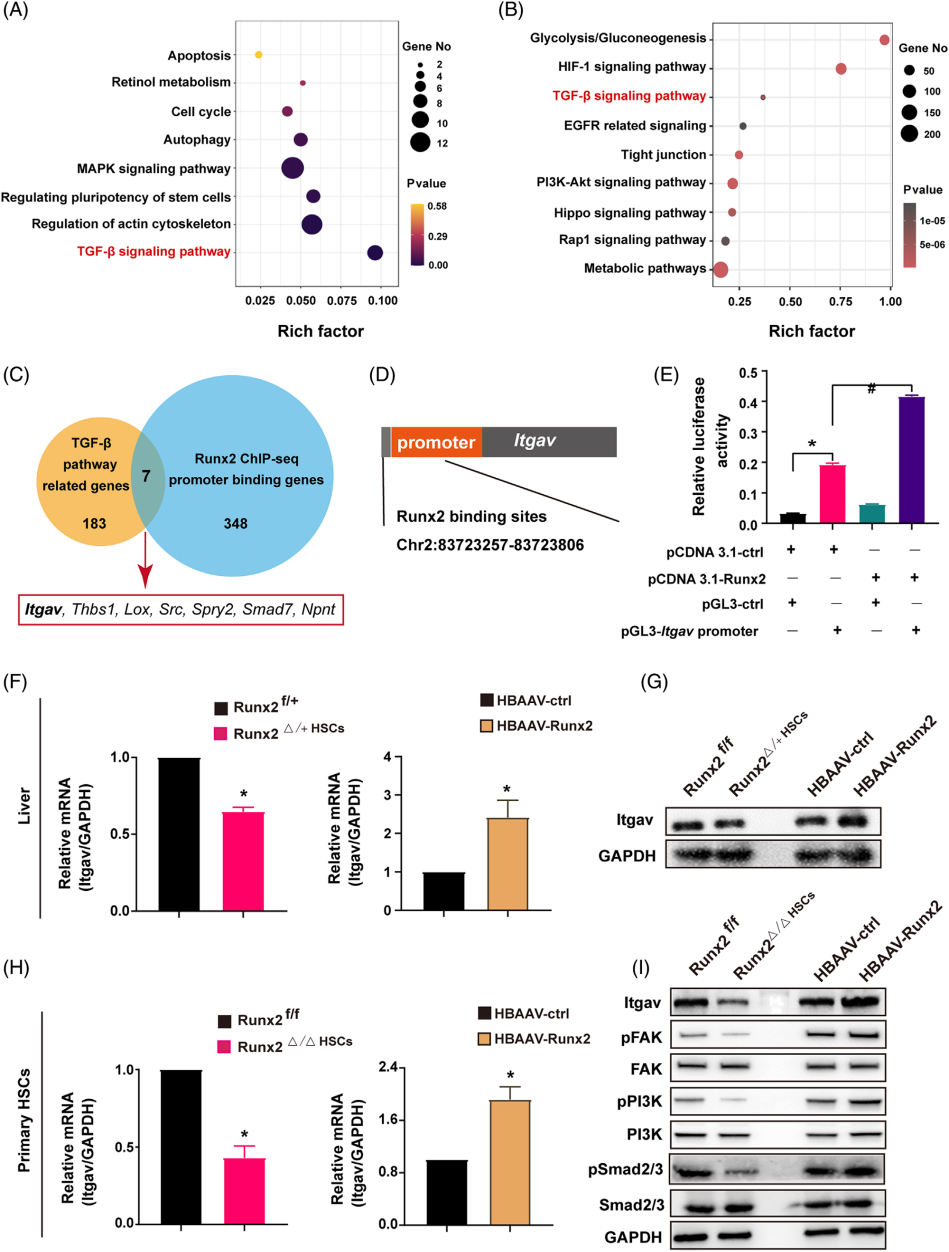
*Itgav* is a direct downstream target of Runx2 in liver fibrosis. (A) RNA‐seq analysis was performed on primary HSC transfected with siRNA of Runx2 or Scramble and cultured for 3 days, and KEGG pathway enrichment analysis of differently expressed genes (DEGs) was performed. (B) Runx2 ChIP‐seq analysis was performed on primary HSC isolated from *HBAAV‐Runx2* mice, and KEGG pathway enrichment analysis of DEGs was conducted. (C) Cross‐analysis of 348 genes whose promoters were bonding with Runx2 according to ChIP‐seq analysis and 183 TGF‐β pathway‐related genes. (D) Genome mapping showed the Runx2 binding sites (Chr2:83723257‐83723806) of the Itgav transcription promoter region. (E) Luciferase reporter analysis was performed on a mouse HSC cell line transfected with pCDNA 3.1‐ctrl or pCDNA 3.1‐Runx2. (F and G) Liver tissues were collected from *Runx2^f/+^*, *Runx2* ***^△^^/+^^HSC^***, *HBAAV‐ctrl* mice or *HBAAV‐Runx2* mice treated with CCl_4_ for 4 weeks. The mRNA and protein expression of Itgav was measured by qRT‐PCR and Western blot assay. (H and I) Primary HSC were isolated from *Runx2^f/f^* mice, *Runx2^△/△HSC^* mice, *HBAAV‐ctrl* mice or *HBAAV‐Runx2* mice. The mRNA expression of Itgav was measured by qRT‐PCR. The protein expression of Itgav and its downstream kinase (FAK, pFAK, PI3K, pPI3K, Smad2/3 and pSmad2/3) were detected by the Western blot assay. Data are mean ± SEM; *n* = 3; ^*^p < .05 versus controls; *^#^p* < .05.

To explore the underlying mechanisms of Runx2 regulating HSC activation, we performed RNA‐sequencing (RNA‐seq) by using primary HSC transfected with siRNA of Runx2. The results exhibited that Runx2 knockdown was mainly associated with impairment of the HSC‐activated signalling pathway, including apoptosis, cell cycle, autophagy, MAPK signalling pathway and TGF‐β signalling pathway according to KEGG pathway enrichment analysis (Figure 6A). As Runx2 acts as a transcriptional factor in various biological processes, the HSC from *HBAAV‐Runx2* mice were then subjected to chromatin immunoprecipitation sequencing (ChIP‐seq) analysis to identify the direct targets of Runx2 in the activated HSC. The results showed that Runx2 bound to numerous DNA sequences with many peaks localised near the transcription start site of target genes (Figure S13). According to KEGG analysis, regions occupied by Runx2 were in proximity of genes related to the phosphatidylinositol‐3‐kinase (PI3K)−Akt signalling pathway, metabolic pathways, TGF‐β signalling pathway, and so forth (Figure 6B). RNA‐seq and ChIP‐seq demonstrated that Runx2 had significant influences on TGF‐β signalling pathway. However, TGF‐β ligands, including TGF‐β1, were not the direct downstream factor of Runx2, indicating Runx2 may regulate the TGF‐β signalling pathway by not directly mediating TGFβ ligands expression.

As the TGF‐β signalling pathway is one of the most important signals in regulating HSC activation and liver fibrosis process,^26^ the TGFβ pathway‐related genes transcriptome and ChIP‐seq datasets were integrated, and seven common genes including integrin alpha‐V (*Itgav*), *Thbs1*, *Lox*, *Src*, *Spry2*, *Smad7* and *Npnt* were obtained (Figure 6C). *Itgav* encodes a major component of αv integrins, which are the key integrins in regulating TGF‐β1 activation and liver fibrosis progression,^20^, ^27^ suggesting that Runx2 might activate HSC via *Itgav* signalling pathway. Runx2 was found to be bound to Chr2:83723257‐83723806 upstream of the transcription start site of *Itgav* using ChIP‐seq analysis, and this binding was confirmed by a luciferase reporter assay (Figure 6D,E). Furthermore, mRNA and protein expression of Itgav in primary HSC or liver tissues from *Runx2^△/△HSC^* mice, *Runx2^△/+HSC^* mice and *HBAAV‐Runx2* mice were examined, and the results demonstrated that Runx2 deficiency or overexpression decreased or increased the expression of *Itgav* in vitro and in vivo (Figure 6F–I).

Given the fact that *Itgav* binds to the ECM ligands and activates downstream kinase, including focal adhesion kinase (FAK) and PI3K, as well as interacting with latent TGF‐β1 to activate TGF‐β1 signalling,^27^, ^28^ we detected the expression of phosphorylated FAK, PI3K and Smad2/3 in primary HSC of Runx2 deficiency or overexpression mice. The results indicated that the expression of phosphorylated FAK, PI3K and Smad2/3 was enhanced in the Runx2 overexpression HSC, while reduced in Runx2 knockdown HSC (Figure 6I). Collectively, our results strongly suggested that Runx2 directly upregulates *Itgav* expression by binding to its promoter, and activates the underlying signalling transduction, which in turn contributes to HSC activation and liver fibrosis progression.

Meanwhile, the matrix showed that *Runx2* is highly correlated with *Itgav* (*r* = .98), *TGF‐β1* (*r* = .91) and *collagen I* (*r* = .97). *Itgav* is also highly correlated with *TGF‐β1* (*r* = .87) and *collagen I* (*r* = .94). These results suggested that there may be some functional relationships between these genes and that they may be co‐regulated or involved in similar biological pathways (Figure S14 and Table S6).

### 3.7 αv Integrins inhibition blocks the aggravation of CCl_4_‐induced liver fibrosis caused by Runx2 overexpression

**FIGURE 7:**
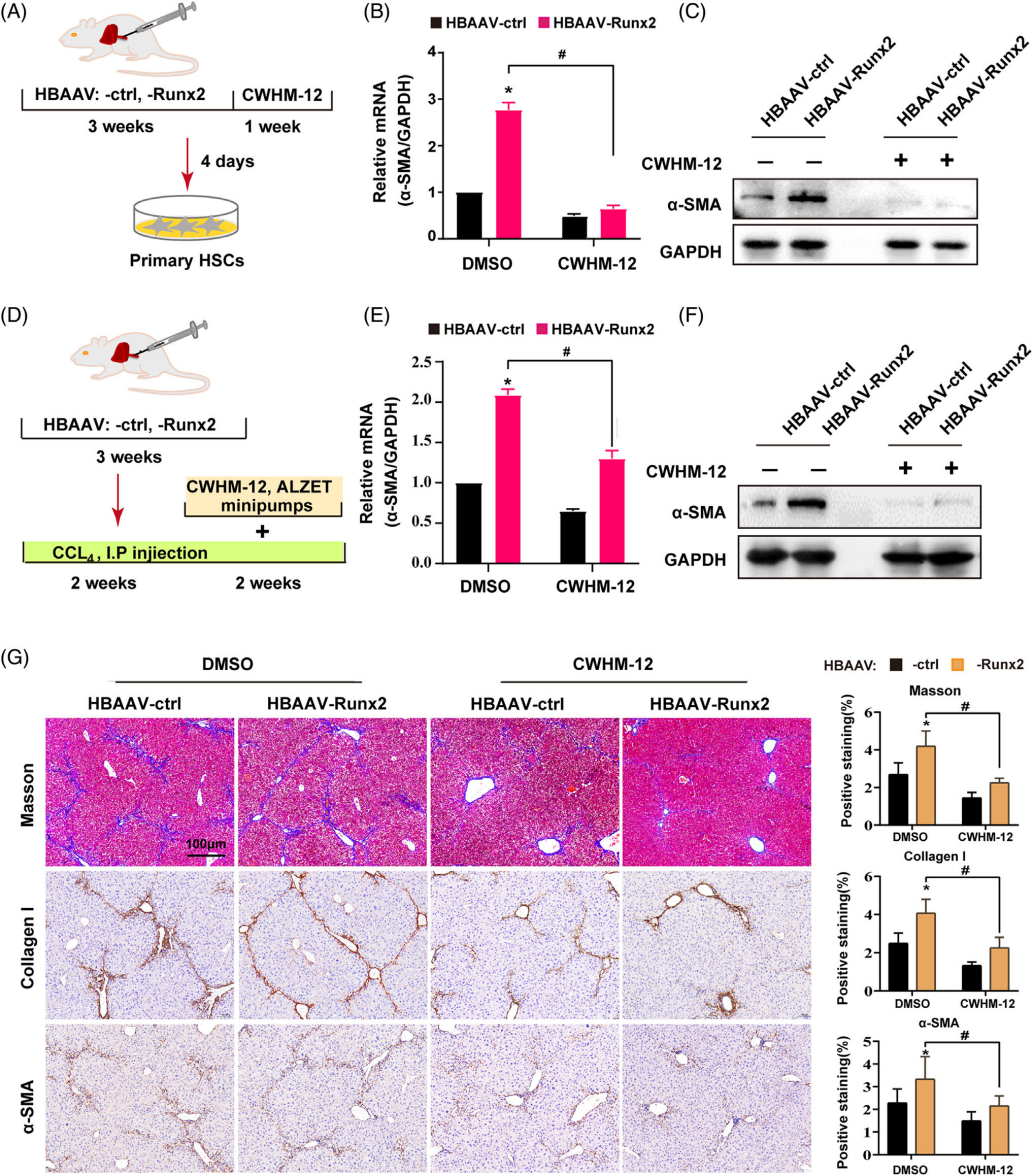
αv Integrins inhibitor blocks the aggravation of CCl_4_‐induced liver fibrosis caused by Runx2 overexpression. (A) The regimen of targeted delivery of αv integrins inhibitor CWHM 12 for in vitro experiments. *HBAAV‐ctrl* mice or *HBAAV‐Runx2* mice were given CWHM‐12 (100 mg/kg/day) or vehicle treatment for 1 week, then the primary HSC were isolated. (B and C) Western blot and qRT‐PCR assays were performed to detect the protein and mRNA expressions of α‐SMA in primary HSC (*n* = 3). (D) The regimen of targeted delivery of CWHM‐12 for in vivo experiments. Mice were given CCl_4_ for 2 weeks, then Alzet minipumps containing either CWHM‐12 (100 mg/kg/day) or vehicle were inserted, followed by a further 2 weeks of CCl_4_. (E and F) The mRNA and protein expressions of α‐SMA in liver tissues from *HBAAV‐Runx2* or *HBAAV‐ctrl* mice treated with or without CWHM‐12 were detected by qRT‐PCR and Western blot assay (*n* = 3). (G) Representative photomicrographs of Masson and IHC staining of collagen I and α‐SMA. Quantification of positive staining areas was measured by ImageJ software. Scale bars: 100 μm (*n* = 5). Data are mean ± SEM; *p < .05 versus controls; ^#^ *p* < .05.

To explore the role of Itgav in Runx2‐related HSC activation and liver fibrosis, we first silenced Itgav by using small interference RNA in primary HSC isolated from HBAAV‐ctrl mice and HBAAV‐Runx2 mice, and the data showed that Itgav knockdown significantly blocked the upregulation of α‐SMA induced by Runx2 overexpression (Figure S15). As the integrin alpha‐V chain, encoded by Itgav, is a major component of five αv integrins, and its expression variation will affect the function of all the five αv integrins,^29^ we thus further utilised a small molecule inhibitor of αv integrins (CWHM‐12) to identify whether Itgav‐mediated function of αv integrins is indeed downstream of Runx2 in HSC activation and liver fibrosis progress (Figure 7A). We found that αv integrins inhibition significantly blocked the upregulation of α‐SMA caused by Runx2 overexpression, which is similar to siItgav (Figure 7B,C). Then, the HBAAV‐Runx2 mice or HBAAV‐ctrl mice were injected with CCl_4_ for 2 weeks to establish liver fibrosis, followed by CWHM‐12 or DMSO administration for another 2 weeks (Figure 7D). The results showed that CWHM‐12 markedly decreased the mRNA and protein expression of α‐SMA and blocked Runx2‐upregulated α‐SMA expression, which was consistent with our in vitro data (Figure 7E,F). Histologically, CWHM‐12 significantly reduced liver fibrosis measured by collagen deposition (Masson staining and collagen I staining) and α‐SMA staining, and importantly, αv integrins inhibition significantly blocked Runx2‐aggravated liver fibrosis (Figure 7G). We also blocked *Itgav* expression in HSC specifically by synchronously intravenously injecting vitamin A‐coupled liposomes carrying *Itgav*‐siRNA (VA‐Lip‐siItgav) and VA‐Lip‐Runx2 in mice for avoiding the broad effect of CWHM‐12, which revealed that *siItgav* inhibited Runx2‐enhanced hepatic fibrosis significantly as well (Figure S16). Therefore, our findings suggested that Runx2 activates HSC and promotes liver fibrosis mainly through *Itgav*.

### 3.8 Activation and nuclear translocation of Runx2 are regulated by PKA in HSC

**FIGURE 8:**
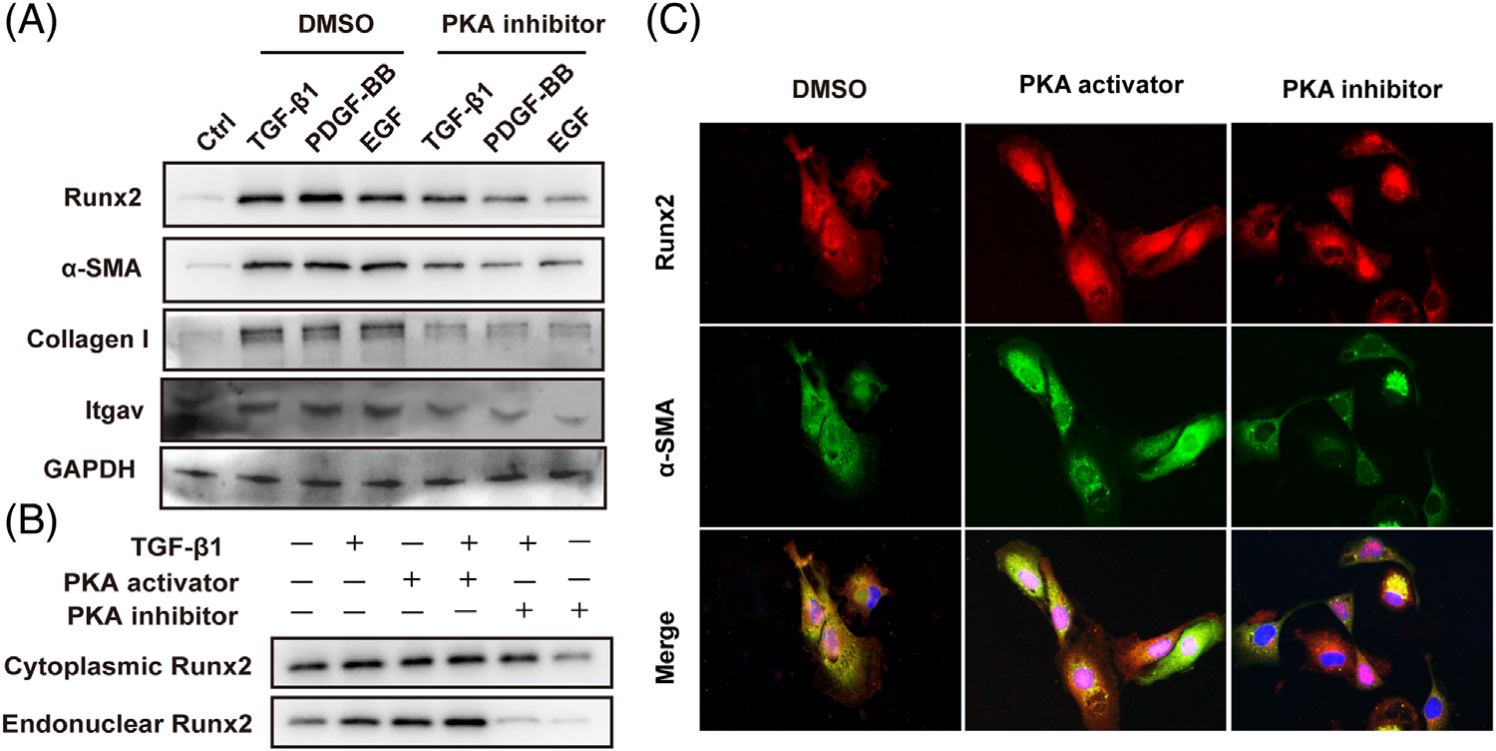
PKA mediated Runx2 activation and nuclear translocation in HSC. (A) Western blot assay of Runx2, α‐SMA, collagen I and Itgav in primary HSC treated with or without TGF‐β1 (5 ng/mL), PDGF‐BB (5 ng/mL) or EGF (5 ng/mL), followed by DMSO or PKA inhibitor (PKI‐6‐22, 10 nM/mL) for 12 h (*n* = 3). (B) Western blot assay of cytoplasmic and endonuclear Runx2 in primary HSC treated with or without TGF‐β1, followed by PKA activator (8‐Bromo‐cAMP, .5 nM/mL) or inhibitor (*n* = 3). (C) Immunofluorescent staining of Runx2 and α‐SMA in primary HSC treated with PKA inhibitor or activator (*n* = 3).

Translocation of Runx2 from the cytoplasm to the nucleus is required for its transcriptional activity,^30^ and Runx2 was reported to be provoked in the cytoplasm and translocated into the nucleus during HSC activation in vitro, as described before. Herein, we attempted to figure out the upstream signalling that elevates Runx2 expression and governs Runx2 translocation into the nucleus. Previous studies have shown that protein kinase A (PKA) activates Runx2 and mediates its nuclear translocation during epithelial‐to‐mesenchymal transition of intestinal epithelial cells and the differentiation of osteoblasts.^31^, ^32^ Moreover, PKA is the common downstream kinase of PDGF, EGF and TGF‐β1, which are essential cytokines in regulating HSC activation.^6^ As a consequence, we utilised TGF‐β1, PDGF‐BB or EGF to activate primary HSC treated with or without PKA inhibitor (PKI‐6‐22). The results displayed that TGF‐β1, PDGF‐BB and EGF all significantly increased Runx2 expression, which was abrogated by the PKA inhibitor (Figure 8A). Additionally, Runx2 can activate *Itgav* transcription, so the effect of PKA on Itgav was detected. The results displayed that TGF‐β1, PDGF‐BB and EGF significantly increased Itgav expression, all of which were abrogated by the PKA inhibitor (Figure 8A). Furthermore, we found that PKA activator (8‐Bromo‐cAMP) could provoke Runx2 nuclear distribution in HSC. In contrast, the PKA inhibitor attenuated the nuclear translocation of Runx2 in HSC cultured with or without TGF‐β1 (Figure 8B). Immunofluorescent staining also indicated that HSC treated with PKA inhibitor resulted in suppressed Runx2 expression in the nucleus and a more quiescent HSC phenotype, whereas PKA activator resulted in a higher nuclear distribution of Runx2 and mesenchymal phenotype characteristics of HSC (Figure 8C). Taken together, these findings demonstrated that TGF‐β1, PDGF‐BB or EGF activated Runx2 and promoted its nuclear translocation through PKA signalling.

## 4 DISCUSSION

**FIGURE 9:**
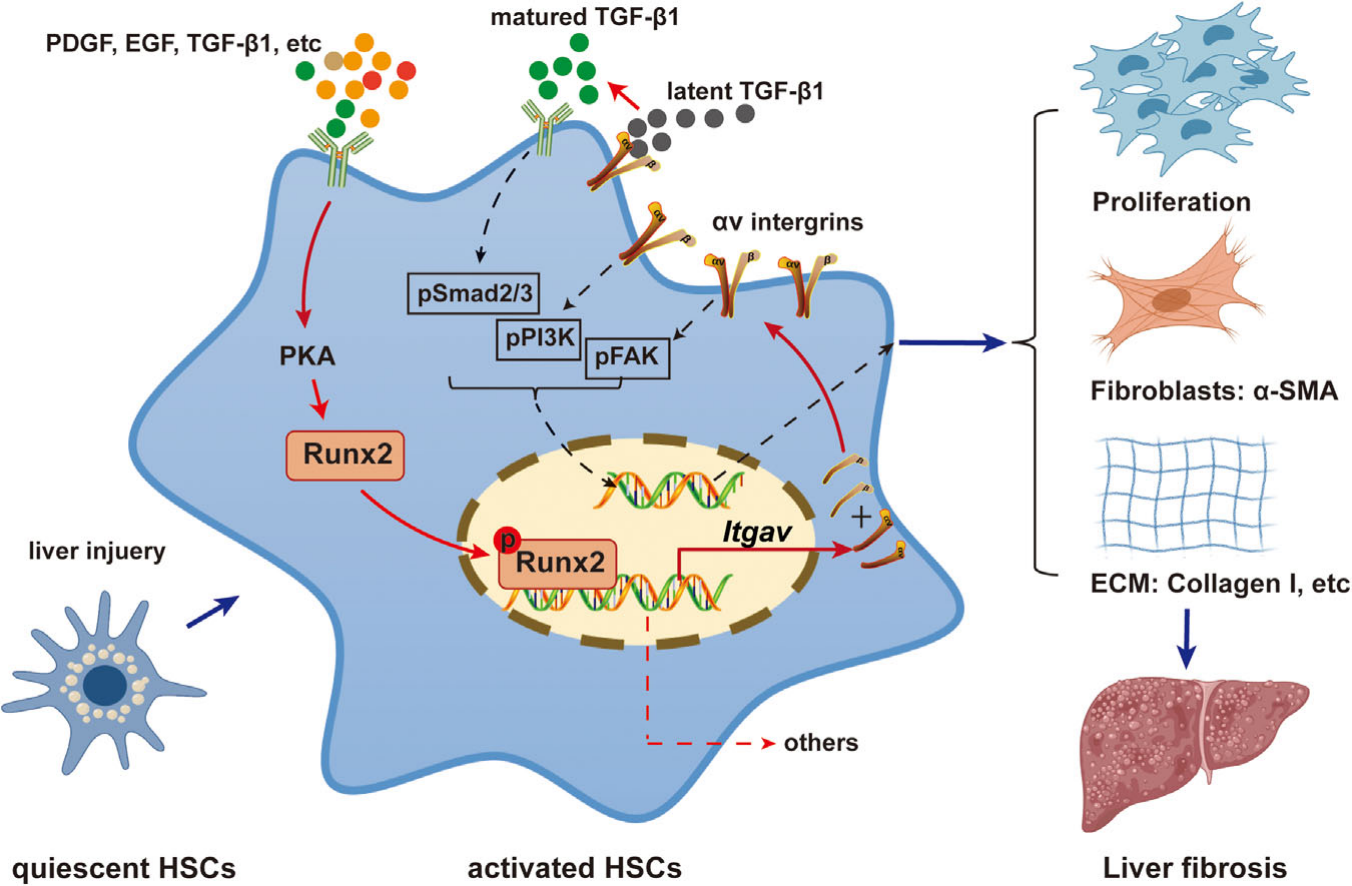
Schematic illustration of Runx2 promoting HSC activation and liver fibrosis process.

Myofibroblasts, which mostly consist of activated HSC in the liver, play a central role in the progression of liver fibrosis.^4^, ^6^ As myofibroblasts are responsible for the excessive synthesis, deposition and remodelling of extracellular matrix proteins, targeting HSC has been thought to be a novel strategy for liver fibrosis management, and increased preclinical studies and clinical trials are being performed on it.^7^, ^21^ In the present study, we demonstrated that Runx2 was specifically expressed in the activated HSC in the liver, and played a critical role during the pathology of liver fibrosis. Our finding may provide a novel target for anti‐fibrotic therapies in the liver. (Figure 9)

We showed that Runx2 was predominantly expressed in nonparenchymal cells in the liver, and subsequent co‐immunofluorescence demonstrated that it was specifically distributed in activated HSC. Runx2 knockdown or overexpression significantly decreased or increased the expression of fibrogenic‐related genes in various HSC, including primary HSC, mouse HSC cell lines and human HSC cell lines, as well as Runx2 regulated HSC proliferation by facilitating the transition from S to G2 stage. HSC‐specific knockdown of Runx2 alleviated CCl_4_‐induced, DDC‐induced or MCD‐induced liver fibrosis, while overexpression of Runx2 by HBAAV‐Runx2 injection exacerbated CCl_4_‐induced liver fibrosis. Taken together, our findings demonstrate that Runx2 facilitates liver fibrosis by promoting HSC activation.

Notably, the expression of Runx2 in quiescent HSC was low, and its upregulation was accompanied by the translocation from cytoplasm to the nucleus during HSC activation. Besides, overexpression of Runx2 did not spontaneously trigger HSC activation or independently cause liver fibrosis in vivo and in vitro, which is consistent with the previous research.^33^ These results indicated that Runx2 is not an initial regulator for HSC activation. Previous studies have shown that TGF‐β1, PDGF and EGF are well‐known stimulators for HSC activation upon liver injury.^4^, ^5^, ^34^ We found that upon TGF‐β1, PDGF and EGF stimulation, the expressions of Runx2, α‐SMA, Itgav and collagen I were significantly elevated in HSC and can be suppressed by the blockade of PKA, which is a family of enzymes mediating a wide variety of cellular functions.^35^ PKA was found to be a vital downstream kinase upon the stimulation of TGF‐β1, PDGF and EGF, and it could activate Runx2 by promoting phosphorylation and translocation of Runx2 in osteoblasts, cancer cells or epithelial cells,^31^, ^36^, ^37^, ^38^, ^39^ which is consistent with our results that PKA agonist and antagonist notably induced or reduced Runx2 nuclear translocation in HSC. Interestingly, TGF‐β1‐, PDGF‐ and EGF‐induced elevation of Runx2 could not be fully blocked by PKA inhibitor treatment, suggesting that there are other mechanisms in which TGF‐β1‐, PDGF‐ and EGF‐activating Runx2 expression exist. Collectively, our results demonstrated that cytokines (TGF‐β1, PDGF, EGF) promoted Runx2 upregulation and nuclear translocation partly through PKA in HSC.

According to the RNA‐seq and ChIP‐seq analysis, Runx2 influenced diversified signalling pathways in HSC, including TGF‐β signalling pathway, autophagy, MAPK signalling pathway and metabolic pathways. It is known that the TGF‐β1 signalling pathway is crucial in regulating HSC activation and liver fibrosis process.^40^ Typically, TGF‐β1 is released in a latent form that must be activated extracellularly, and many activation mechanisms are involved in the regulation.^41^, ^42^ Surprisingly, Runx2 did not regulate TGF‐β1 signalling directly, but was bound to the promoter of *Itgav*, which plays a vital role in the activation of latent TGF‐β1.^29^ Additionally, Runx2 deficiency or overexpression decreased or increased the expression of Itgav and its downstream kinases (pFAK, pPI3K and pSmad2/3), which indicates that Runx2 activates the Itgav signalling pathway.

Integrins are composed of α/β heterodimers, which comprise 18 different α subunits and eight β subunits. Itgav is the αv subunit that combines with five different β subunits, including β1, β3, β5, β6 and β8, forming αv integrins.^29^ The αv integrins are the master regulators during fibrosis due to their role in the control of TGF‐β1 activity by releasing matured TGF‐β1 from latent TGF‐β1.^43^, ^44^ Previous studies showed that selective deletion of Itgav in HSC protected CCl_4_‐induced liver fibrosis,^20^, ^44^ suggesting that Runx2 transcriptionally upregulating Itgav expression may be responsible for the progression of liver fibrosis in our study. Interestingly, individual deletion of β subunit partners of Itgav was unable to effectively inhibit mice liver fibrosis induced by CCl_4_, indicating a combinatory effect of all the αv integrins to drive fibrosis, which strongly suggests that inhibiting pan‐αv integrins may be required to obtain significant anti‐fibrotic effects.^20^, ^27^, ^29^ Expectedly, CWHM‐12, a novel small molecule inhibitor therapeutically targeting all αv integrins effectively, was shown to significantly reduce liver fibrosis,^20^, ^45^ which was also observed in our study, and more importantly, CWHM‐12 could prevent the liver fibrosis aggravation caused by Runx2 overexpression. However, the majority of the pan‐αv integrin inhibitors are designed based on Arg‐Gly‐Asp (RGD) structure in the latent TGF‐β1, which shares the same structure with other ligands that can also be recognised by αv integrins, such as fibronectin,^46^ suggesting that blocking αv integrins by RGD structure may cause some unwanted side effects. As a consequence, targeting the Runx2‐*Itgav* axis may be another choice of pan‐αv integrins inhibiting for liver fibrosis treatment. Notably, as blocking αv integrins could not fully rescue Runx2 overexpression accelerating liver fibrosis, we could not exclude the possibility that other downstream targets are involved in Runx2‐mediated HSC activation and liver fibrosis.

Taken together, our findings demonstrated that Runx2 is a crucial regulator participating in liver fibrosis by activating HSC. Importantly, PKA/Runx2/Itgav is a probable mechanism for regulating HSC activation. Targeting Runx2 may represent a promising target for establishing therapeutic strategies for liver fibrosis.

## CONFLICT OF INTEREST STATEMENT

The authors declare they have no conflicts of interest.

## Supporting information

Supporting Information

Supporting Information

Supporting Information

Supporting Information

Supporting Information

Supporting Information

Supporting Information

Supporting Information

Supporting Information

Supporting Information

Supporting Information

Supporting Information

Supporting Information

Supporting Information

Supporting Information

Supporting Information

Supporting Information

Supporting Information

Supporting Information

Supporting Information

Supporting Information

Supporting Information

Supporting Information
