# Supplementary figures and images for: Runx2 activates hepatic stellate cells to promote liver fibrosis via transcriptionally regulating Itgav expression
Source: Clin Transl Med. 2023 Jul 5;13(7):e1316. doi: 10.1002/ctm2.1316 (PMC10320748; doi:10.1002/ctm2.1316)

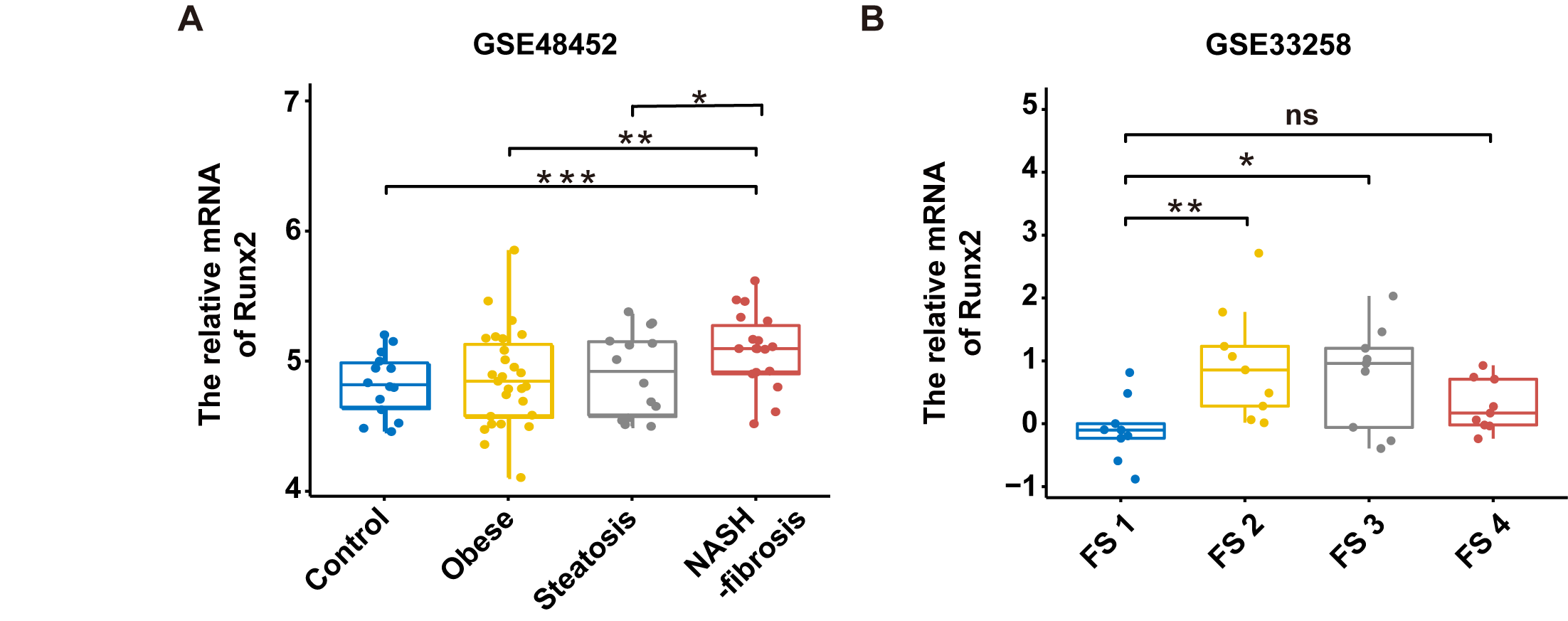

Supplement: Supplementary file 1 — Supporting Information [file CTM2-13-e1316-s012.tif]

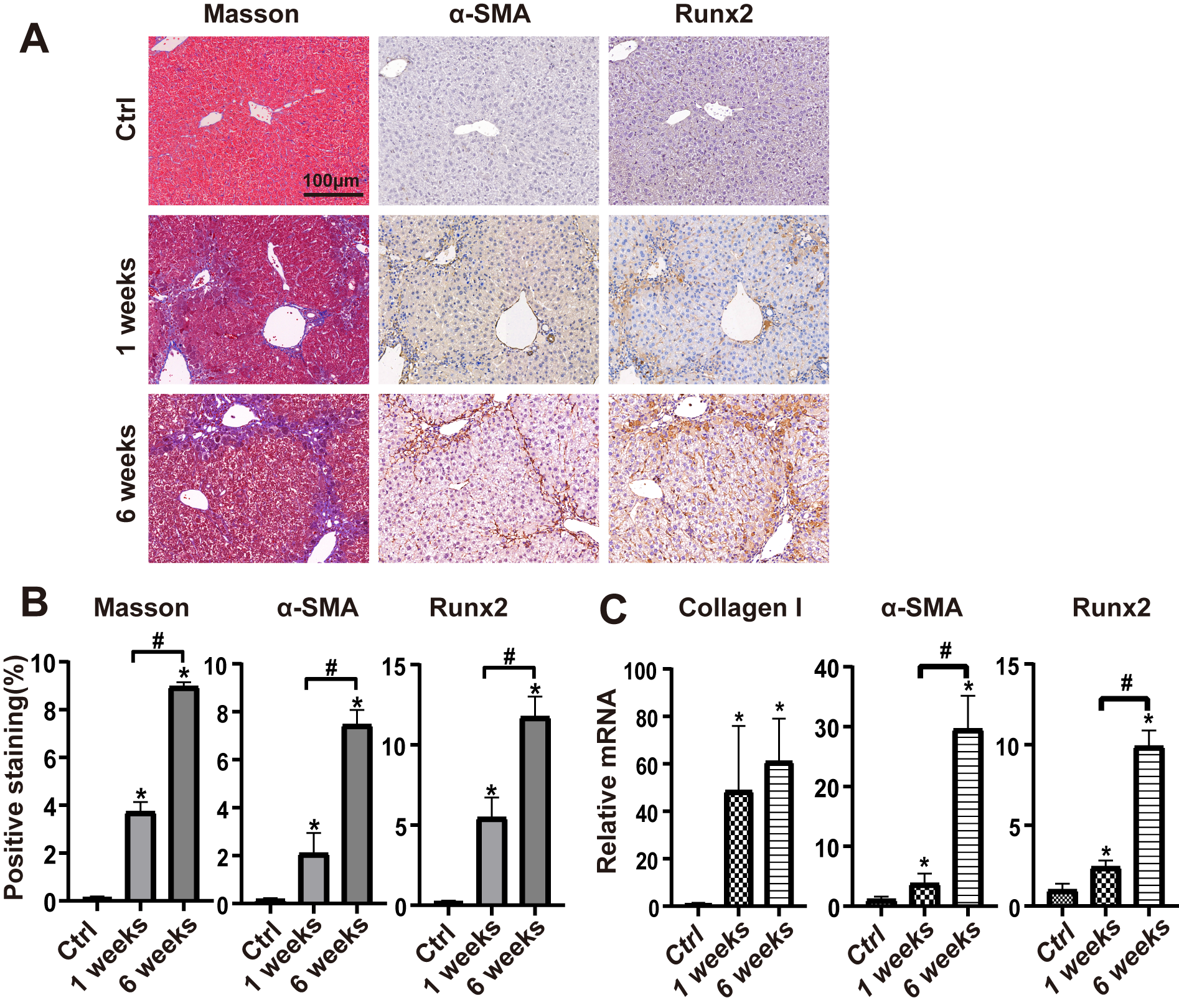

Supplement: Supplementary file 2 — Supporting Information [file CTM2-13-e1316-s008.tif]

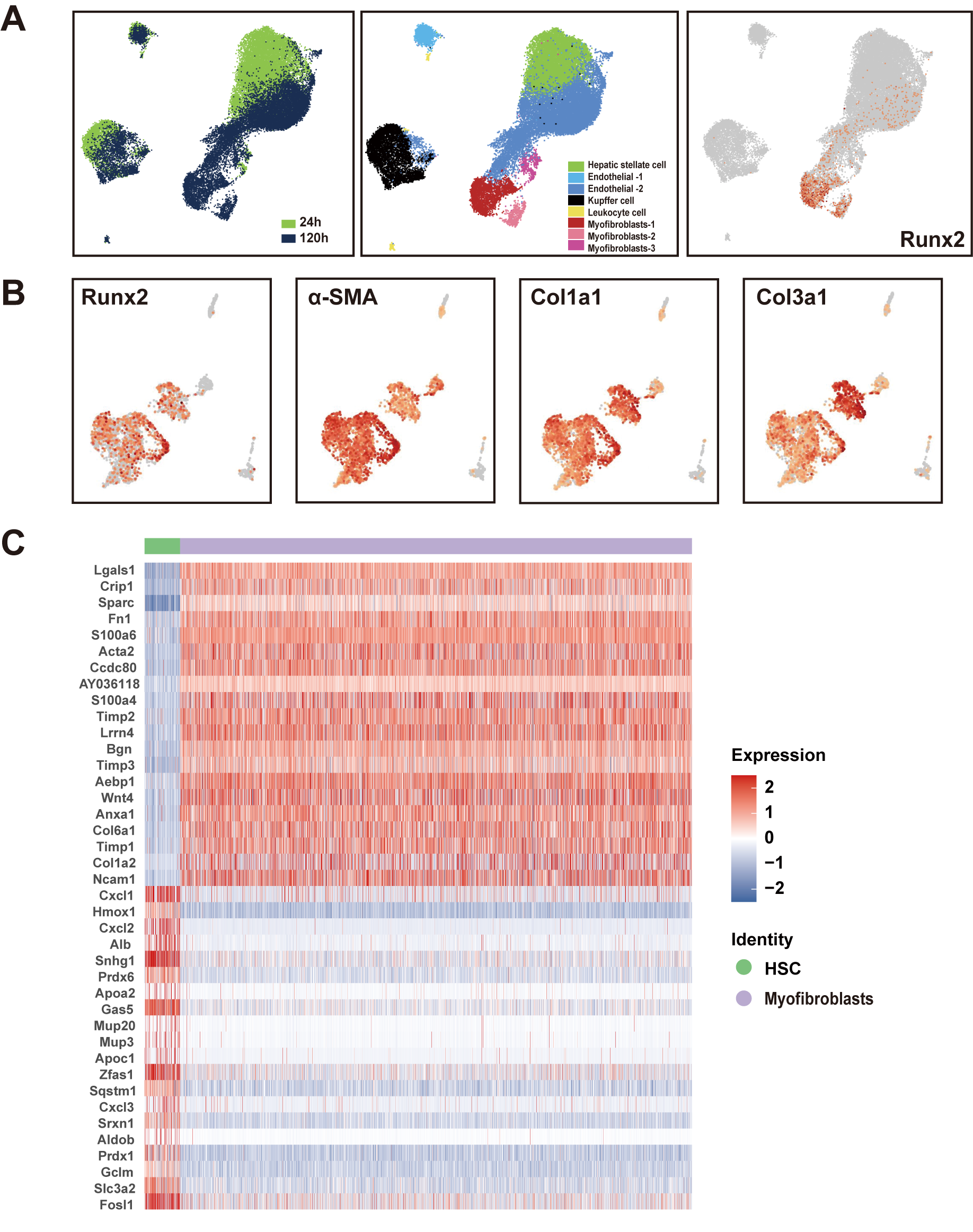

Supplement: Supplementary file 3 — Supporting Information [file CTM2-13-e1316-s001.tif]

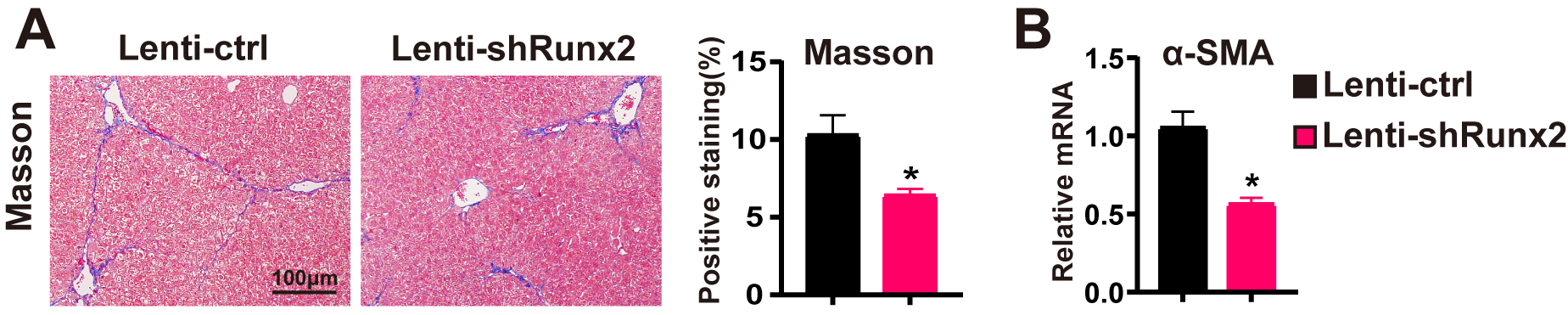

Supplement: Supplementary file 4 — Supporting Information [file CTM2-13-e1316-s016.tif]

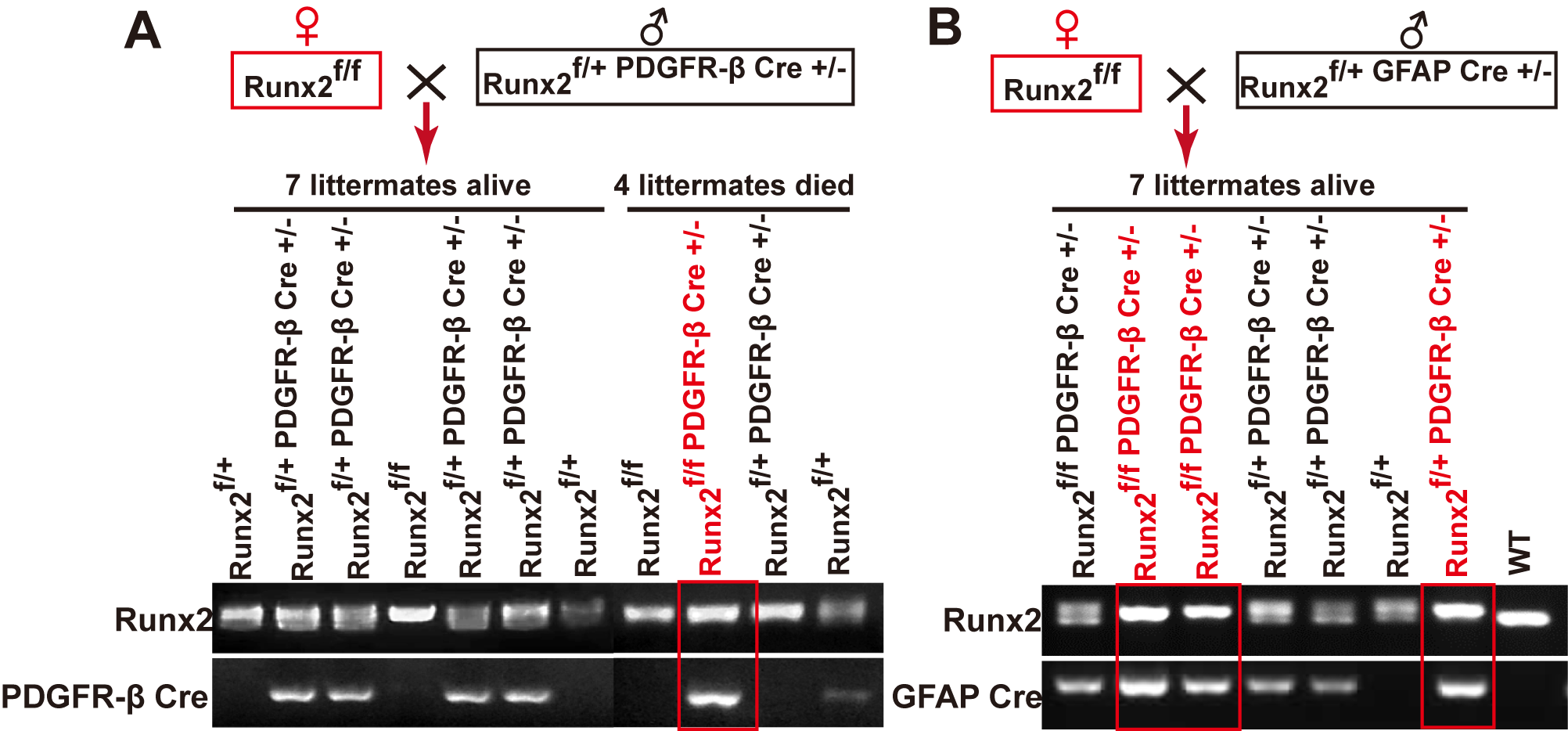

Supplement: Supplementary file 5 — Supporting Information [file CTM2-13-e1316-s002.tif]

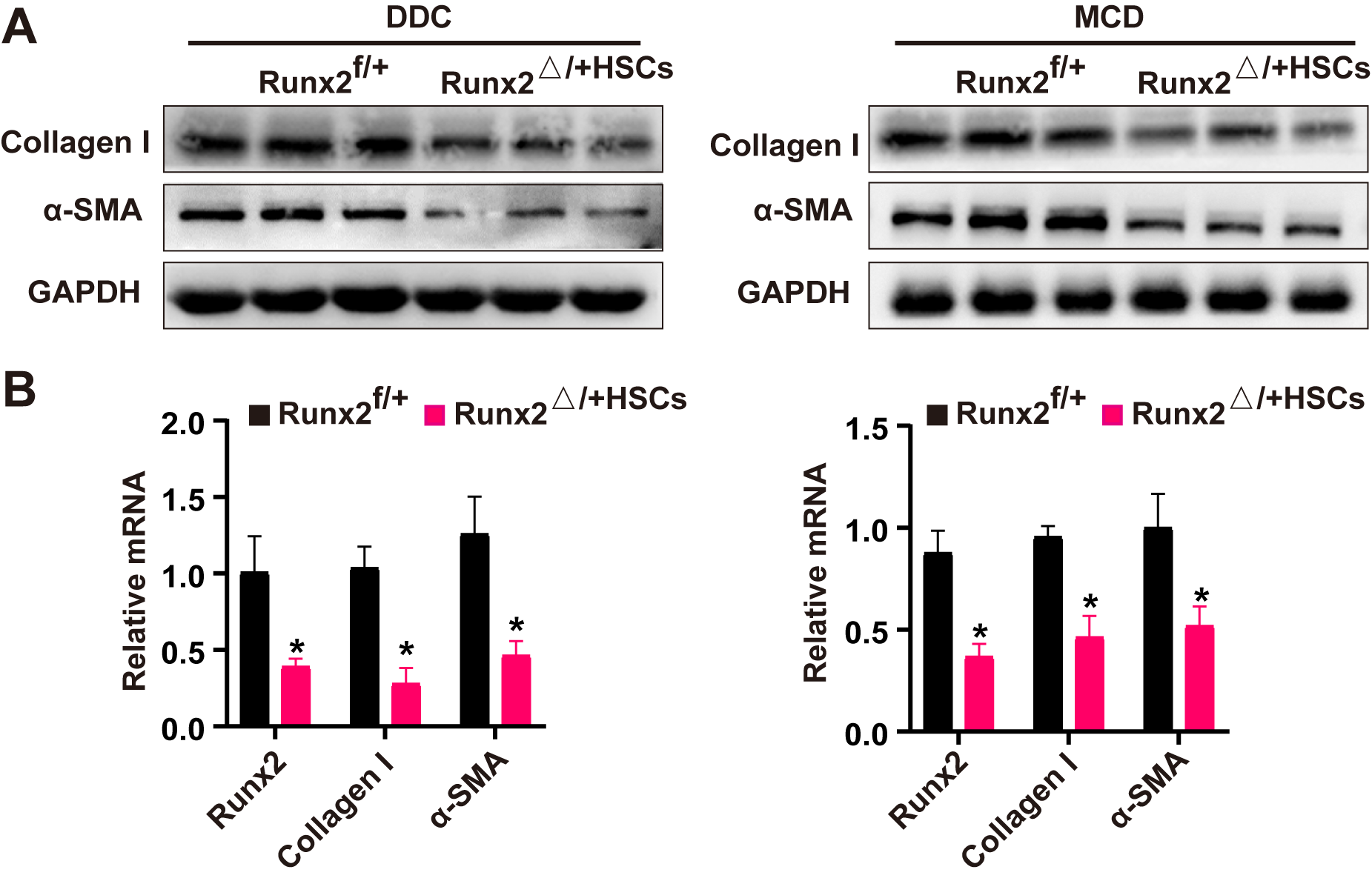

Supplement: Supplementary file 6 — Supporting Information [file CTM2-13-e1316-s004.tif]

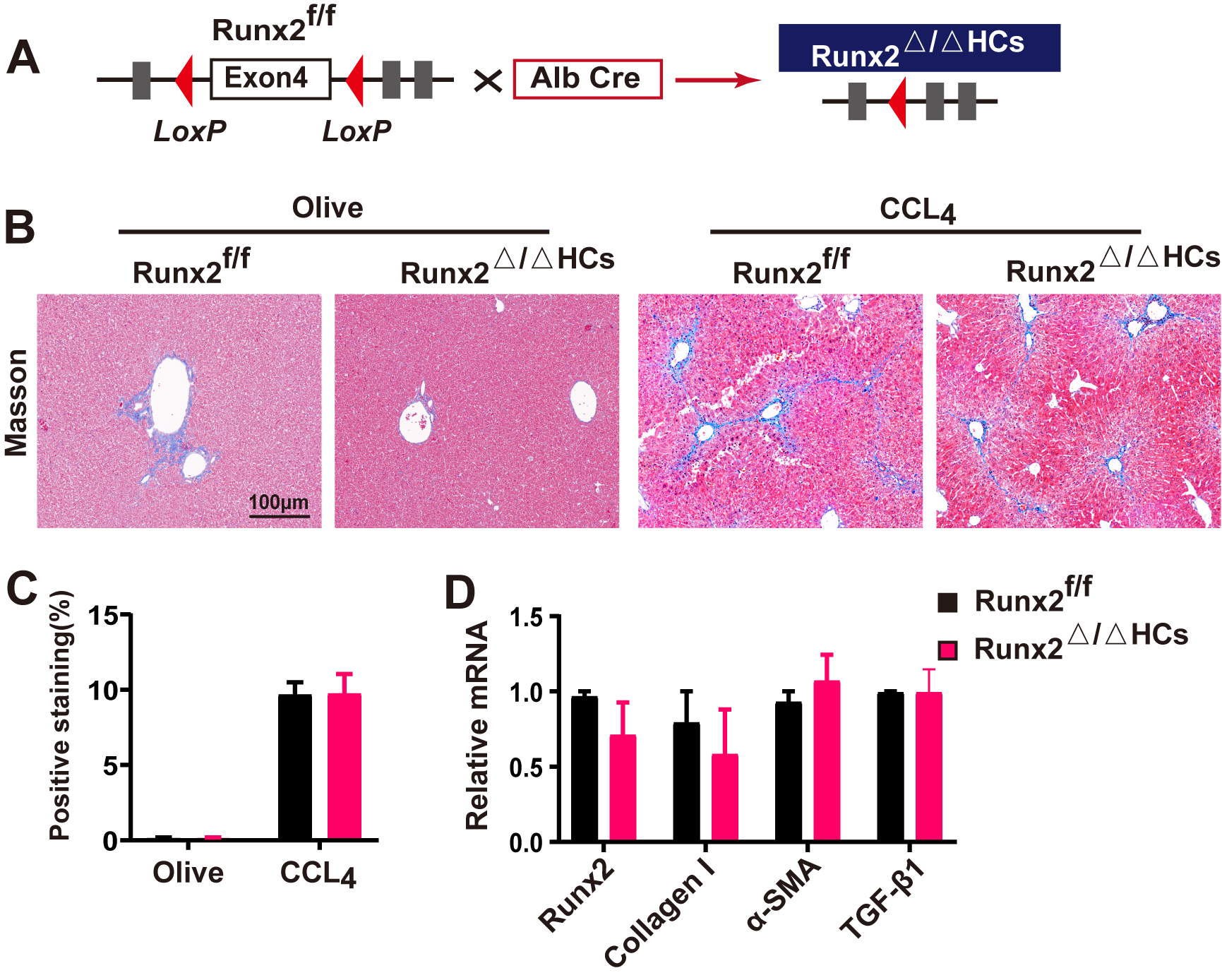

Supplement: Supplementary file 7 — Supporting Information [file CTM2-13-e1316-s014.tif]

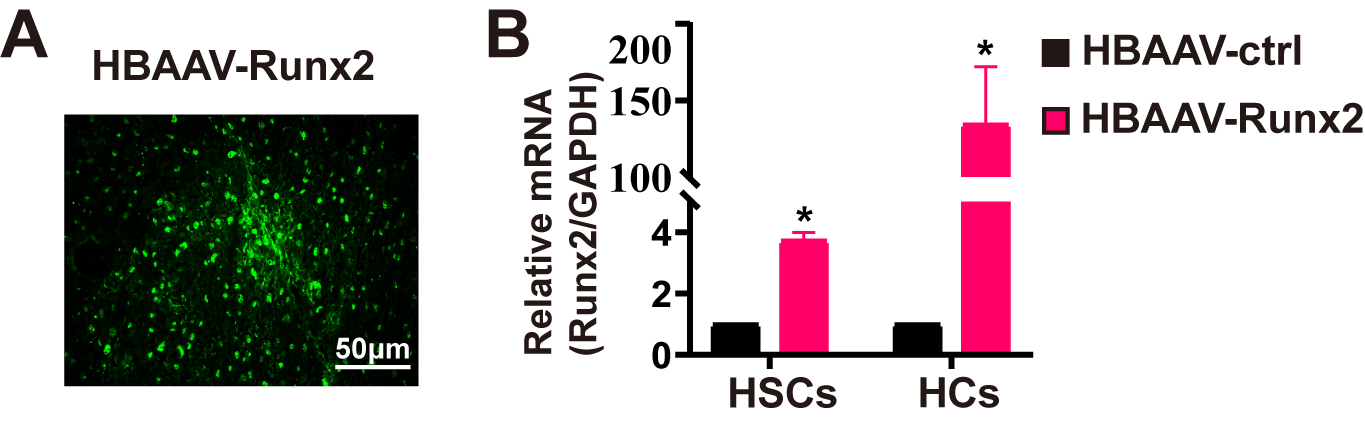

Supplement: Supplementary file 8 — Supporting Information [file CTM2-13-e1316-s019.tif]

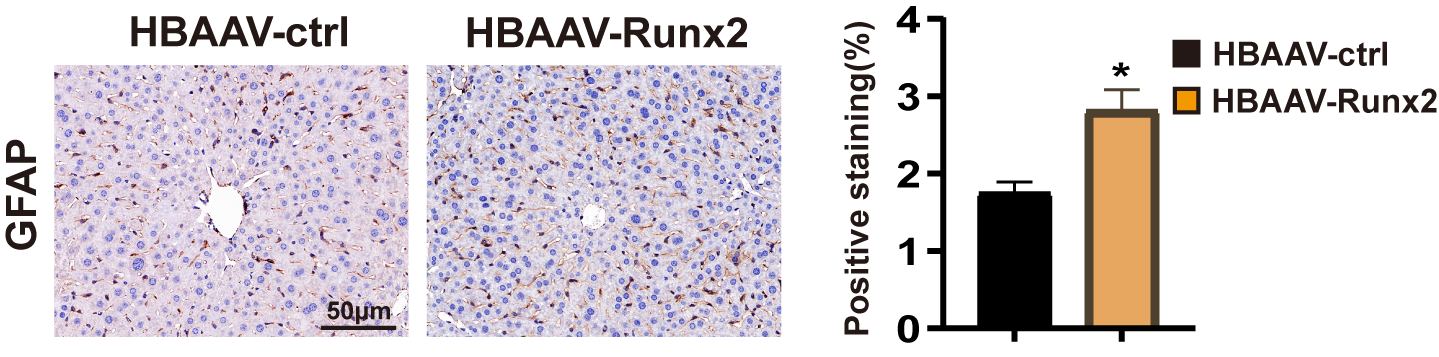

Supplement: Supplementary file 9 — Supporting Information [file CTM2-13-e1316-s010.tif]

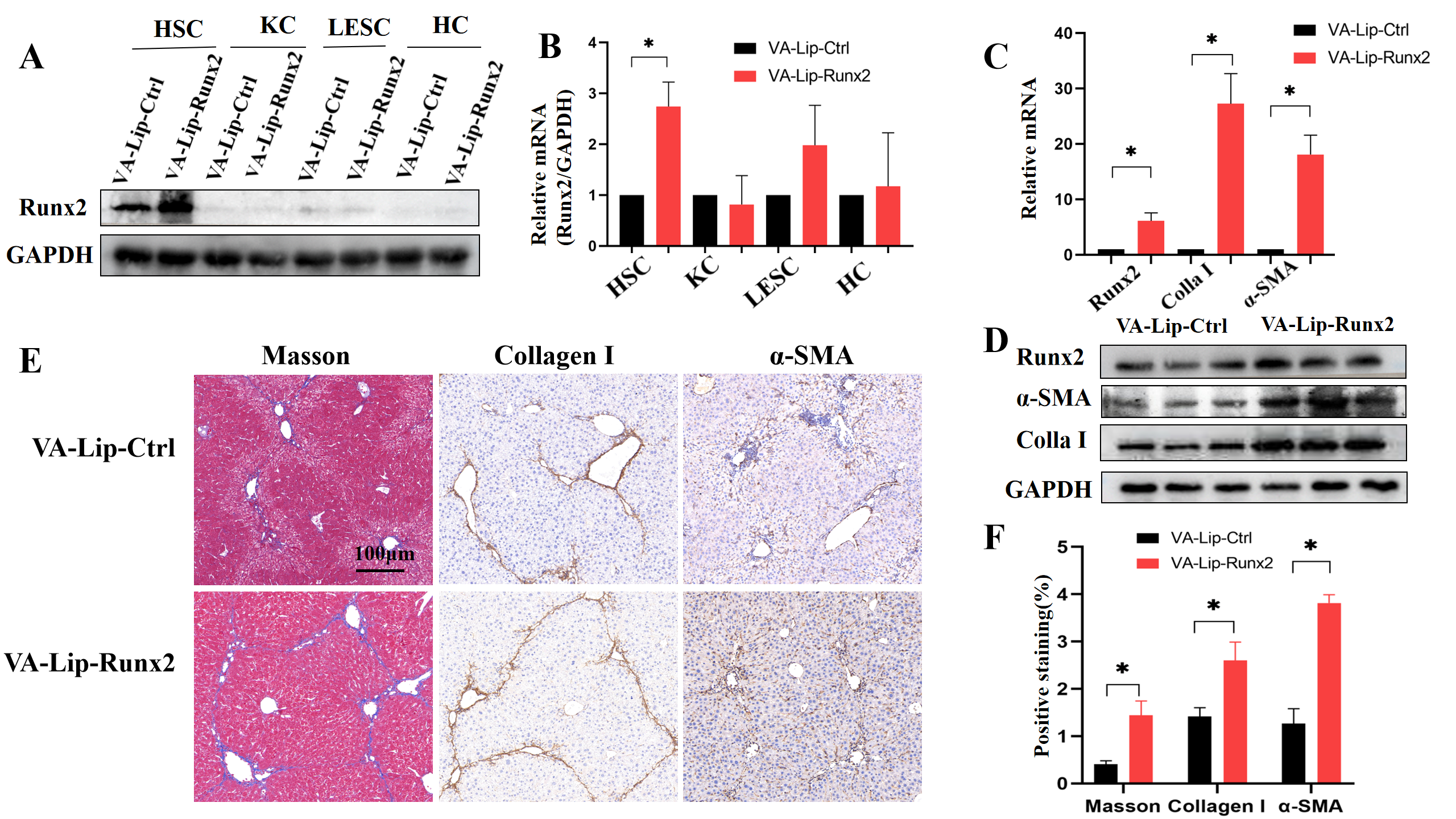

Supplement: Supplementary file 10 — Supporting Information [file CTM2-13-e1316-s022.tif]

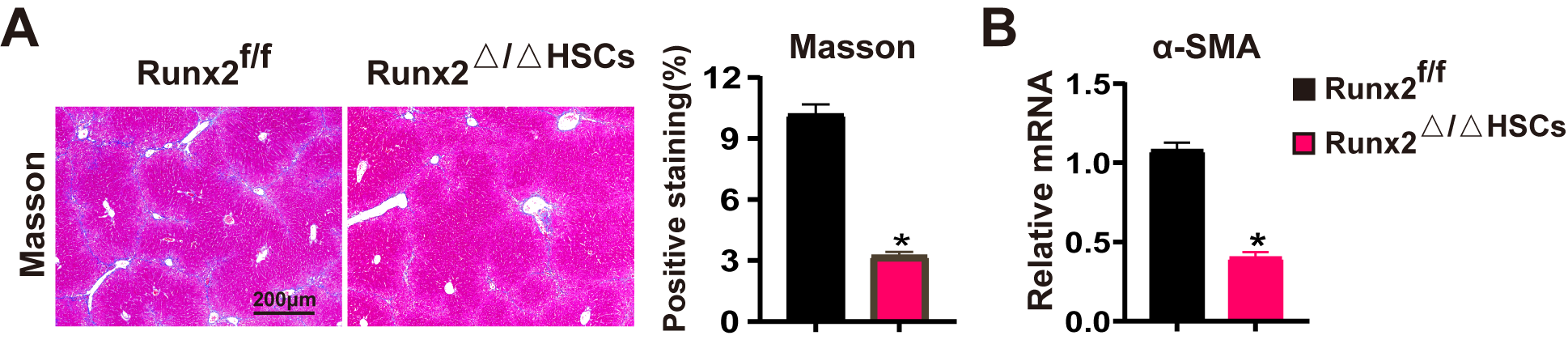

Supplement: Supplementary file 11 — Supporting Information [file CTM2-13-e1316-s009.tif]

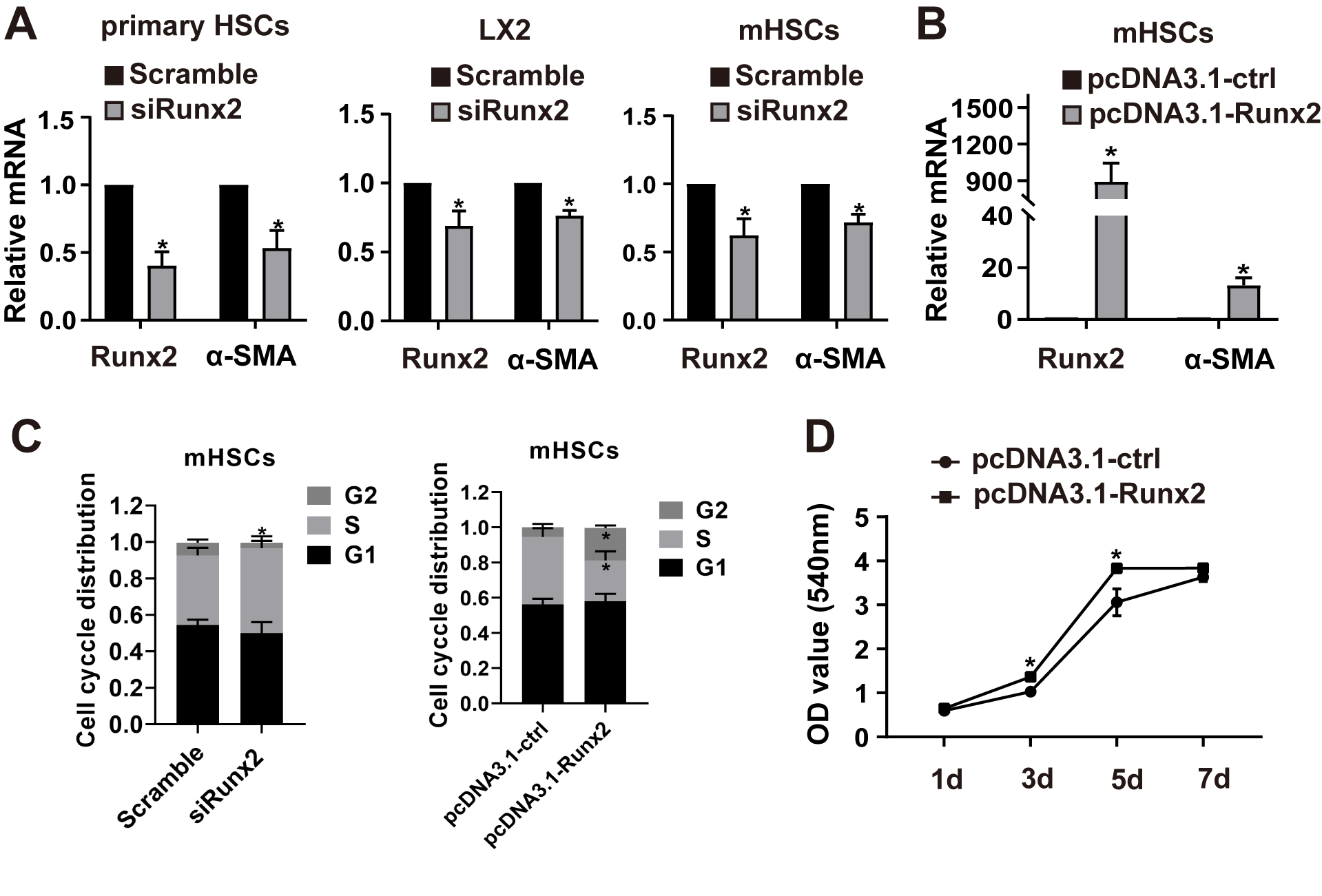

Supplement: Supplementary file 12 — Supporting Information [file CTM2-13-e1316-s018.tif]

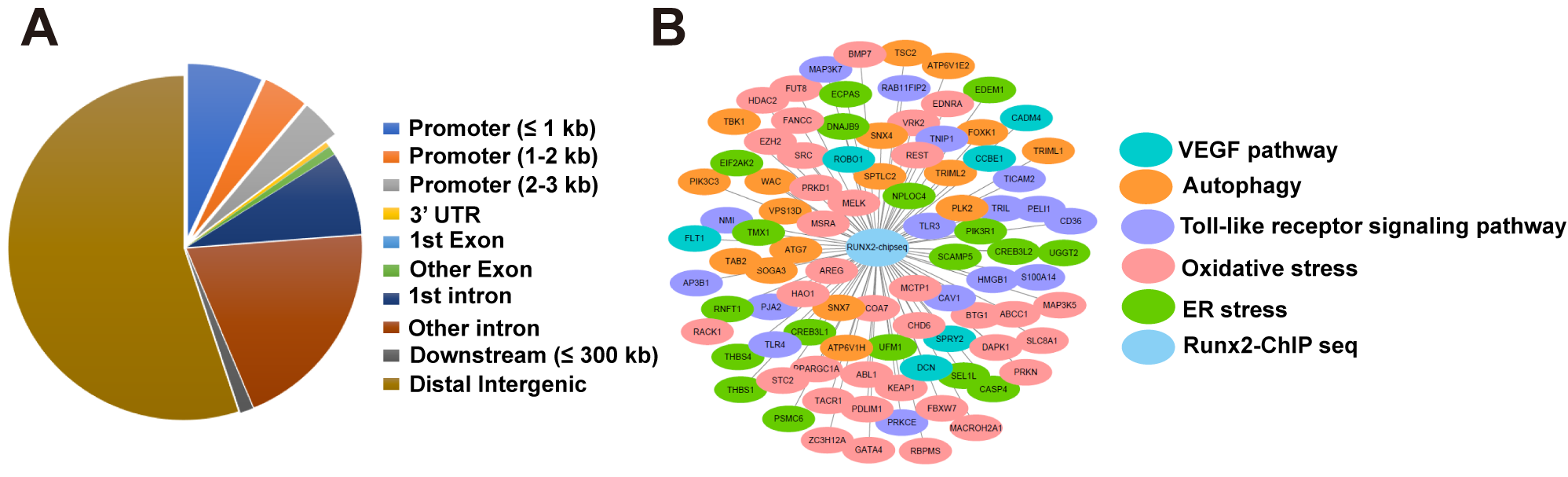

Supplement: Supplementary file 13 — Supporting Information [file CTM2-13-e1316-s005.tif]

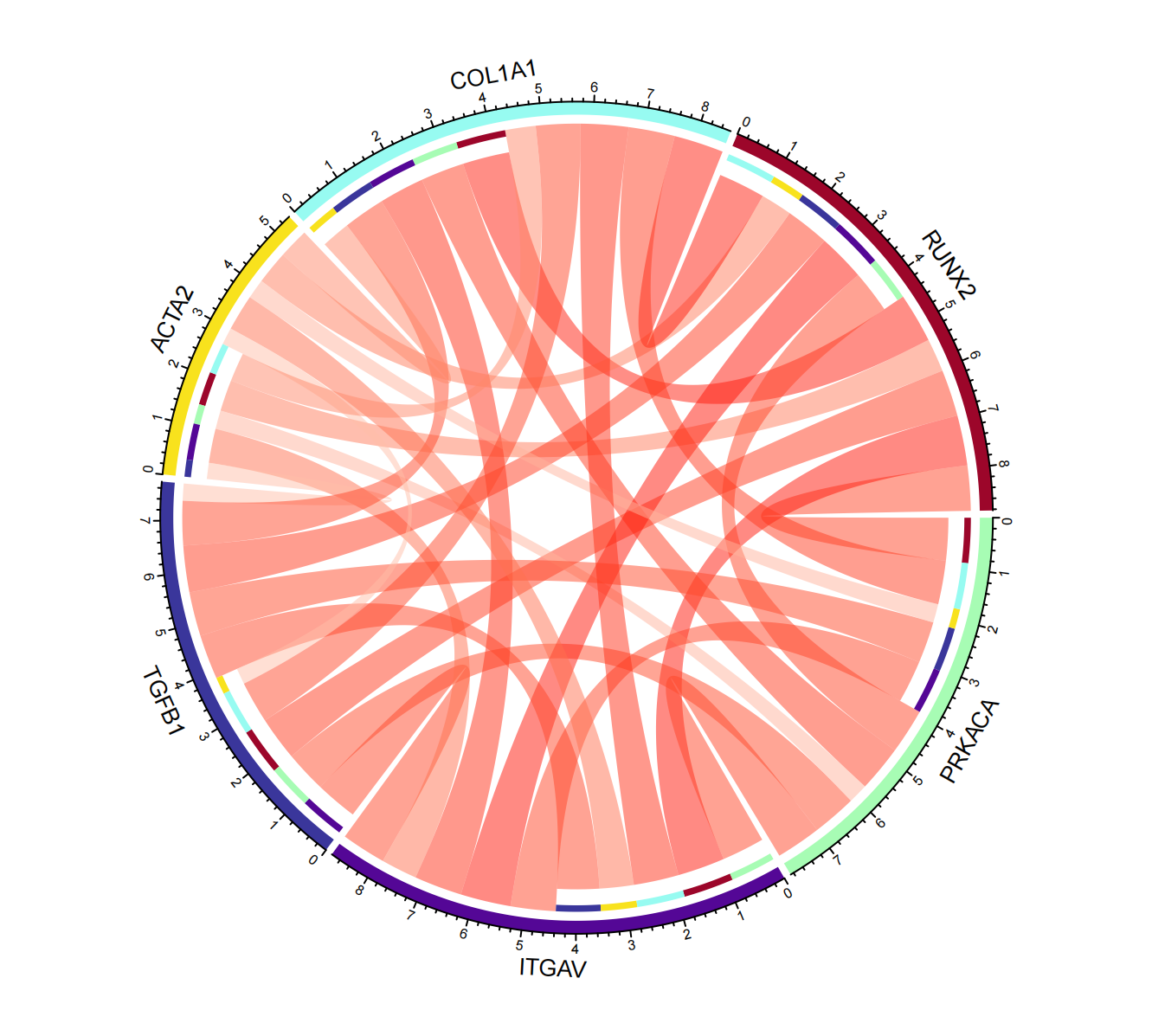

Supplement: Supplementary file 14 — Supporting Information [file CTM2-13-e1316-s003.tif]

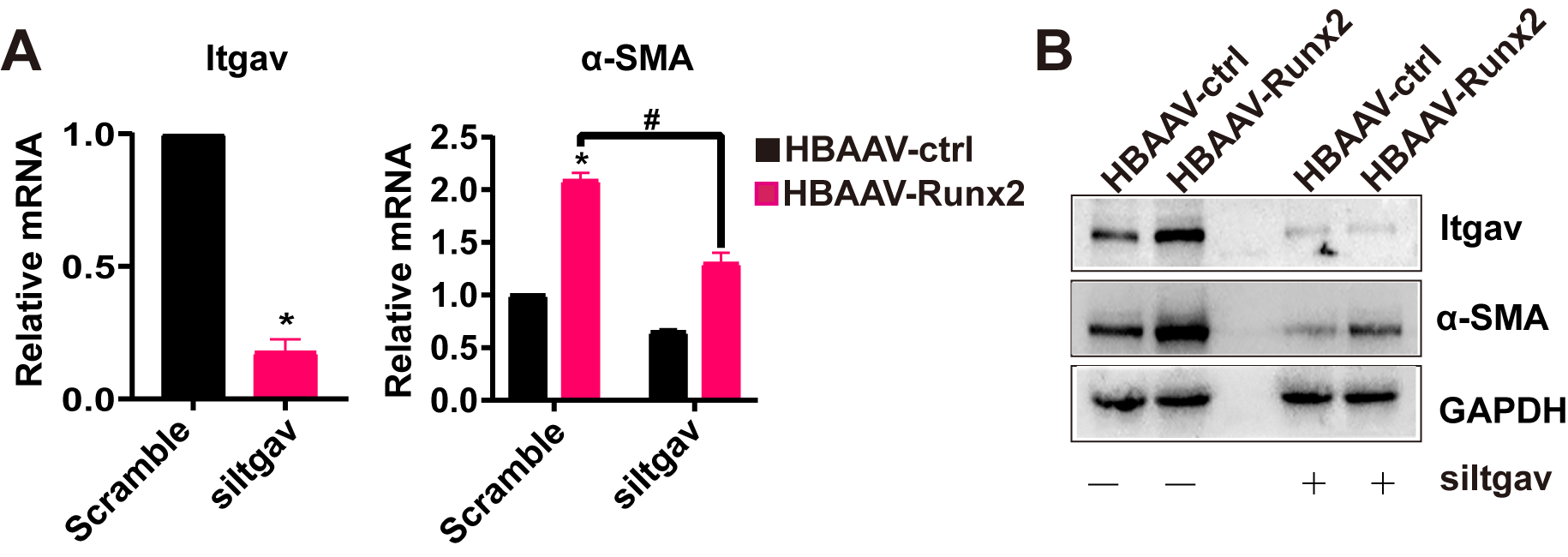

Supplement: Supplementary file 15 — Supporting Information [file CTM2-13-e1316-s013.tif]

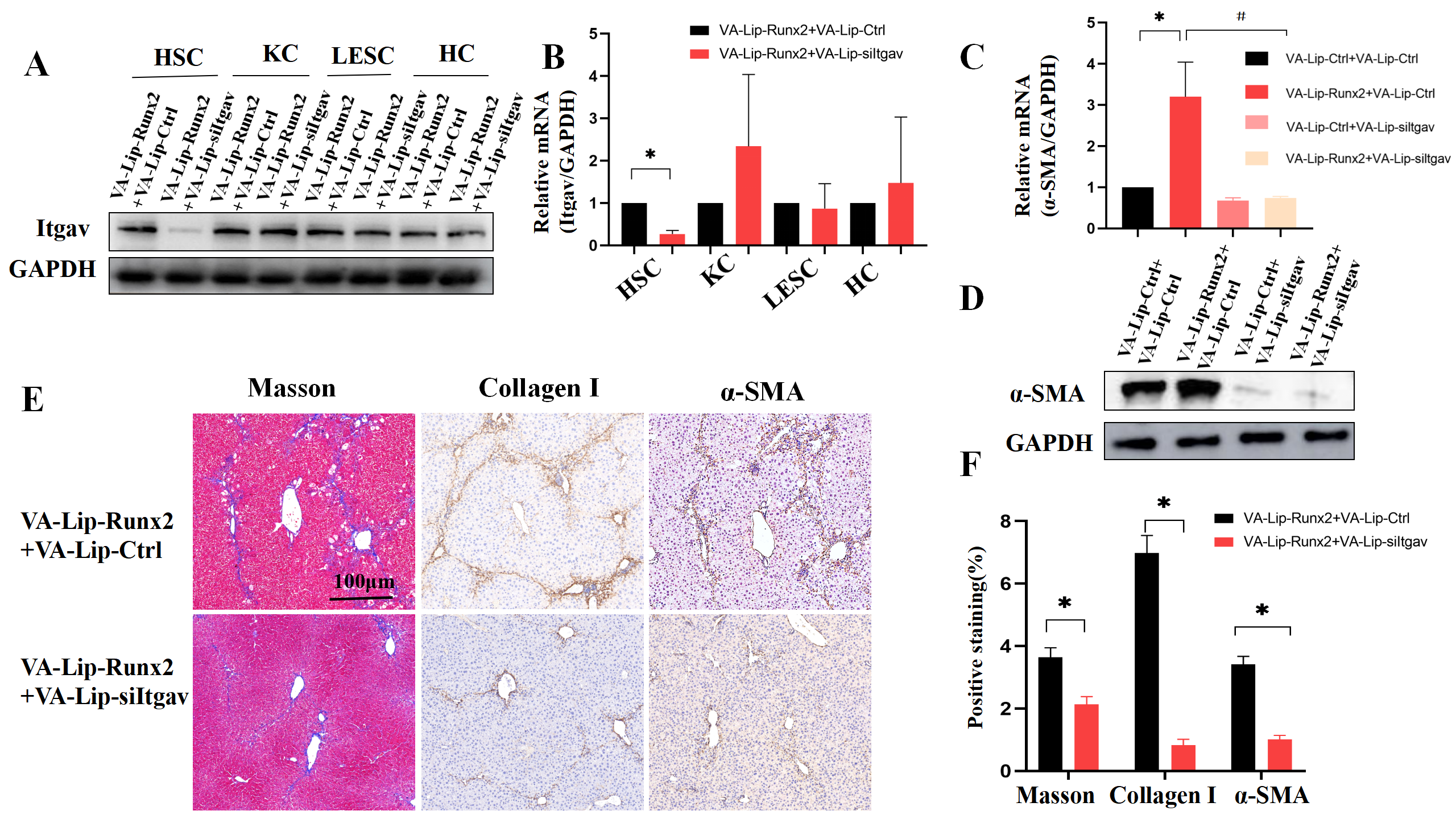

Supplement: Supplementary file 16 — Supporting Information [file CTM2-13-e1316-s006.tif]
