# Supplementary material for: Runx2 activates hepatic stellate cells to promote liver fibrosis via transcriptionally regulating Itgav expression
Source: Clin Transl Med. 2023 Jul 5;13(7):e1316. doi: 10.1002/ctm2.1316 (PMC10320748; doi:10.1002/ctm2.1316)
Supplement: Supplementary file 17 — Supporting Information [file CTM2-13-e1316-s011.docx]

| **Table S1. Information of patients with liver fibrosis.** | | | | |
| --- | --- | --- | --- | --- |
| **ID** | **Age** | **Sex** | **Etiology** | **Statement** |
| 1 | 42 | Female | PBC | G2，S3 |
| 2 | 47 | Female | PBC | G2，S4 |
| 3 | 51 | Female | PBC+AIH | G2，S3 |
| 4 | 53 | Female | PBC+AIH | G4，S4 |
| 5 | 38 | Male | HBV | G2，S4 |
| 6 | 33 | Male | HBV | G1，S3 |
| 7 | 61 | Male | PBC+NASH | G2，S3 |
| 8 | 49 | Female | HSOS | G0，S4 |
| 9 | 36 | Male | HCV | G1，S4 |
| 10 | 43 | Female | HCV | G1，S4 |

**Abbreviations:** PBC, primary biliary cholangitis；AIH, autoimmune hepatitis; HBV, hepatitis B virus; HCV, hepatitis C virus; NASH, non-alcoholic steatohepatitis; HSOS, hepatic sinusoidal obstructive syndrome
