# Supplementary material for: Runx2 activates hepatic stellate cells to promote liver fibrosis via transcriptionally regulating Itgav expression
Source: Clin Transl Med. 2023 Jul 5;13(7):e1316. doi: 10.1002/ctm2.1316 (PMC10320748; doi:10.1002/ctm2.1316)
Supplement: Supplementary file 18 — Supporting Information [file CTM2-13-e1316-s021.docx]

| **Table S2. Genotyping primer sequences were presented as follows:** | |
| --- | --- |
| Primers | Sequence (5’-3’) |
| *Runx2 f/f* | F: CCTGGGATAAGCGCGTTAGG  R: GGCCTAGCTGCTCAGGAGAAC |
| *GFAP Cre* | F: TAGCCCACTCCTTCATAAAGCCCT  R: GCTAAGTGCCTTCTCTACACC |
| *PDGFRβ Cre* | F: GTTCGCAAGAACCTGATGGACA R: CTAGAGCCTGTTTTGCACGTTC |
| *Alb Cre* | F: TGTAGAGAAG GCACTTAGC  R: CCAGGTTACGGATATAGTTCA |
| F: Forward; R: reverse. | |
|  | |
