# Supplementary material for: Runx2 activates hepatic stellate cells to promote liver fibrosis via transcriptionally regulating Itgav expression
Source: Clin Transl Med. 2023 Jul 5;13(7):e1316. doi: 10.1002/ctm2.1316 (PMC10320748; doi:10.1002/ctm2.1316)
Supplement: Supplementary file 19 — Supporting Information [file CTM2-13-e1316-s023.docx]

| **Table S3. siRNA and shRNAs sequences, plasmid primers.** | |
| --- | --- |
| Name | Sequence (5’-3’) |
| Mouse shRunx2 | GCCCAGGCGTATTTCAGATGA |
| Mouse siRunx2 | F: GCUUCUCCAACCCACGAAUTT  R: AUUCGUGGGUUGGAGAAGCTT |
| Mouse siItgav | F: GACCCGUUGUCACUGUAAATT  R: UUUACAGUGACAACGGGUCTT |
| Human siRunx2 | F: GGCACAGACAGAAGCUUGATT  R: UCAAGCUUCUGUCUGUGCCTT |
| AAV-m-Runx2-Bam/Kpn | F: gacctccatagaagacaccgggatccGCCACCATGGC  GTCAAACAGCCTCTT  R: ccttgtagtcgttaattaaggtaccATATGGCCGCCAA  ACAGACT |
| Mouse pcDNA3.1-Runx2 | F: gctagcATGGCGTCAAACAGCCTCTTC  R: ctcgagTCAATATGGCCGCCAAACAGAC |
| Mouse pGL3-Itgav promoter | F: gctagcGGCTCAGGGGGGCCTACTG  R: ctcgagGGCCTCTGTGTGTATCAGGC |
| F: Forward; R: reverse | |
