# Supplementary material for: Runx2 activates hepatic stellate cells to promote liver fibrosis via transcriptionally regulating Itgav expression
Source: Clin Transl Med. 2023 Jul 5;13(7):e1316. doi: 10.1002/ctm2.1316 (PMC10320748; doi:10.1002/ctm2.1316)
Supplement: Supplementary file 20 — Supporting Information [file CTM2-13-e1316-s007.docx]

| **Table S4. The antibodies used in the study.** | | | | |
| --- | --- | --- | --- | --- |
| **Antibody** | **Catalog No** | **Company** | **Application** | **Dilution** |
| Runx2 | ab236639 | Abcam, Cambridge, MA | ChIP | 1:40 |
| Runx2 | ab76956 | Abcam, Cambridge, MA | WB, IHC, IF | 1:1000/1:200 |
| Runx2 | D130-3 | MBL, Japan | WB | 1:500 |
| α-SMA | ab5694 | Abcam, Cambridge, MA | WB, IHC, IF | 1:1000/1:300/1:200 |
| α-SMA | GB111364 | Servicebio, China | IHC | 1:500 |
| Collagen I | 72026 | CST, Danvers, MA | WB, IHC | 1:1000/1:300 |
| TGF-β1 | 346599 | Zen Bio Science, China | WB | 1:500 |
| CD31 | GB11063-2 | Servicebio, China | IF | 1:200 |
| F4/80 | GB11027 | Servicebio, China | IF | 1:200 |
| Itgav | 381872 | Zen Bio Science, China | WB | 1:500 |
| GFAP | ab7260 | Abcam, Cambridge, MA | IHC | 1:300 |
| PI3K | 4257 | CST, Danvers, MA | WB | 1:1000 |
| pPI3K(Tyr458) | 17366 | CST, Danvers, MA | WB | 1:1000 |
| FAK | 3285 | CST, Danvers, MA | WB | 1:1000 |
| pFAK | 8556 | CST, Danvers, MA | WB | 1:1000 |
| Smad2/3 | ab202445 | Abcam, Cambridge, MA | WB | 1:1000 |
| pSmad2/3 | ab254407 | Abcam, Cambridge, MA | WB | 1:1000 |
| GAPDH | ab8245 | Abcam, Cambridge, MA | WB | 1:2000 |
| Anti-Mouse | SA00001-1 | Sanying Bioteclmology, China | WB | 1:10000 |
| Anti-Rabbit | SA00001-2 | Sanying Bioteclmology, China | WB | 1:10000 |
| Anti-Mouse | ab150115 | Abcam, Cambridge, MA | IF | 1:200 |
| Anti-Rabbit | ab150075 | Abcam, Cambridge, MA | IF | 1:200 |
| Anti-Mouse | ab150113 | Abcam, Cambridge, MA | IF | 1:200 |
| Anti-Rabbit | ab150077 | Abcam, Cambridge, MA | IF | 1:200 |
