# Supplementary material for: Runx2 activates hepatic stellate cells to promote liver fibrosis via transcriptionally regulating Itgav expression
Source: Clin Transl Med. 2023 Jul 5;13(7):e1316. doi: 10.1002/ctm2.1316 (PMC10320748; doi:10.1002/ctm2.1316)
Supplement: Supplementary file 21 — Supporting Information [file CTM2-13-e1316-s015.docx]

| **Table S5. Real-Time Polymerase Chain Reaction Primers.** | |
| --- | --- |
| Gene | Sequence (5’-3’) |
| Mouse α-SMA | F: GATCTCTATGCTAACAACGTCCTG  R: CTTCGTCGTATTCCTGTTTGC |
| Mouse Col1a1 | F: CTGGCGGTTCAGGTCCAAT  R: TTCCAGGCAATCCACGAGC |
| Mouse Runx2 | F: CAGGAAGACTGCAAGAAGGCTCTGG  R: ACACGGTGTCACTGCGCTGAAGA |
| Mouse TGF-β1 | F: GAGGCGGTGCTCGCTTTGTA  R: CGTTGTTGCGGTCCACCATTA |
| Mouse Desmin | F: AACTACAGGAGGAAATCCAACTAAG  R: CCAGGTCAATACGAGCTAGAGTG |
| Mouse Col3a1 | F: TGGAGACGATCCAATGGATTTC  R: CTTGAGTTCGGGGTGGCAGA |
| Mouse MMP2 | F: AATGCCATCCCTGATAACCTG  R: ATGCTTCCAAACTTCACGCTC |
| Mouse MMP9 | F: GTGGGACCATCATAACATCACATAC  R: GGATGACAATGTCCGCTTCG |
| Mouse MMP13 | F: ATCATACTACCATCCTGCGACTC  R: CTCGGAGCCTGTCAACTGTG |
| Mouse TIMP-1 | F: ATGCTAAAAGGATTCAAGGCTG  R: AACTCTTCACTGCGGTTCTGG |
| Mouse PDGFRβ | F: GGTGTTCGAGGCTTATCCG  R: TCGTGGAAGGCTCGCATAG |
| Mouse Itgav | F: GACTGGATAGAGGCAAGAGCG  R: GCAGATGACTTCAGCGAATAGG |
| Mouse GAPDH | F: AACACGGAAGGCCATGCCAG  R: TGCATCCTGCACCACCAACT |
| Human α-SMA | F: GGCTGTTTTCCCATCCATTG  R: GGCTGTTTTCCCATCCATTG |
| Human Runx2 | F: CCATAACCGTCTTCACAAATCC  R: GTTCCCGAGGTCCATCTACTG |
| Human GAPDH | F: CACTCCTCCACCTTTGACGC  R: CTGTTGCTGTAGCCAAATTCGT |
| F, Forward; R, reverse. | |
