# Supplementary material for: Runx2 activates hepatic stellate cells to promote liver fibrosis via transcriptionally regulating Itgav expression
Source: Clin Transl Med. 2023 Jul 5;13(7):e1316. doi: 10.1002/ctm2.1316 (PMC10320748; doi:10.1002/ctm2.1316)
Supplement: Supplementary file 22 — Supporting Information [file CTM2-13-e1316-s020.docx]

| **Table S6. CORRELATION DATA** | | | | | | |
| --- | --- | --- | --- | --- | --- | --- |
| **GENE** | **RUNX2** | **ITGAV** | **TGFB1** | **ACTA2** | **COL1A1** | **PKA** |
| **RUNX2** | 1.0000000 | 0.9839871 | 0.9140074 | 0.6547347 | 0.9731594 | 0.8847925 |
| **ITGAV** | 0.9839871 | 1.0000000 | 0.8746672 | 0.7103069 | 0.9364784 | 0.8847522 |
| **TGFB1** | 0.9140074 | 0.8746672 | 1.0000000 | 0.3353631 | 0.8697021 | 0.8657255 |
| **ACTA2** | 0.6547347 | 0.7103069 | 0.3353631 | 1.0000000 | 0.5989538 | 0.3875039 |
| **COL1A1** | 0.9731594 | 0.9364784 | 0.8697021 | 0.5989538 | 1.0000000 | 0.9077404 |
| **PKA** | 0.8847925 | 0.8847522 | 0.8657255 | 0.3875039 | 0.9077404 | 1.0000000 |
