# Supplementary material for: Runx2 activates hepatic stellate cells to promote liver fibrosis via transcriptionally regulating Itgav expression
Source: Clin Transl Med. 2023 Jul 5;13(7):e1316. doi: 10.1002/ctm2.1316 (PMC10320748; doi:10.1002/ctm2.1316)
Supplement: Supplementary file 23 — Supporting Information [file CTM2-13-e1316-s017.docx]

**Runx2 activates hepatic stellate cells to promote liver fibrosis via up-regulating αv integrins**

Li Zhong^1,2*^, Jinqiu Zhao^3*^, Lu Huang^4,5,6*^, Yi Liu^2^, Xiaoxiao Pang^7,8^, Ke Zhan^1^, Shan Li^1^, Qian Xue^1^, Xiaoli Pan^9^, Liang Deng^1^

^1^Department of Gastroenterology, The First Affiliated Hospital of Chongqing Medical University, Chongqing, China,

^2^Department of Gastroenterology and Hepatology, The Second Affiliated Hospital of Chongqing Medical University, Chongqing, China,

^3^Department of Infectious Diseases, The FirstAffiliated Hospital of Chongqing Medical University, Chongqing, China

^4^Chongqing Key Laboratory of Child Infection and Immunity, ^5^Department of Pediatric Research Institute, ^6^Ministry of Education Key Laboratory of Child Development and Disorders, Children's Hospital of Chongqing Medical University, Chongqing, China

^7^Chongqing Key Laboratory of Oral Diseases and Biomedical Sciences, ^8^Chongqing Municipal Key Laboratory of Oral Biomedical Engineering of Higher Education, Stomatological Hospital of Chongqing Medical University, Chongqing, China

^9^Department of Gastroenterology, Union Hospital, Tongji Medical College, Huazhong University of Science and Technology, Wuhan, China

Email:

Li Zhong, [305864@hospital.cqmu.edu.cn](mailto:305864@hospital.cqmu.edu.cn); Jinqiu Zhao:jinqiuzhao@hospital.cqmu.edu.cn, Lu Huang, [huanglu_86oct@163.com](mailto:huanglu_86oct@163.com); Yi Liu, [ly61yyy@163.com](mailto:ly61yyy@163.com); Xiaoxiao Pang, [pangxxhi@hospital.cqmu.edu.cn](mailto:pangxxhi@hospital.cqmu.edu.cn); Ke Zhan, [zhanke@hospital.cqmu.edu.cn](mailto:zhanke@hospital.cqmu.edu.cn); Shan Li, [andylee24@foxmail.com](mailto:andylee24@foxmail.com); Qian Xue, [drxueqian@163.com](mailto:drxueqian@163.com); Xiaoli Pan, [pxiaoli@hotmail.com](mailto:pxiaoli@hotmail.com); Liang Deng, [dengliang@cqmu.edu.cn](mailto:dengliang@cqmu.edu.cn).

*These authors contributed equally to this work.

**ADDRESS CORRESPONDENCE AND REP RINT REQUESTS TO:**

Liang Deng, PhD, Department of Gastroenterology, The First Affiliated Hospital of Chongqing Medical University. No 1, Youyi road, Yuanjia gang, Yuzhong district, Chongqing, 400042, China. Email: [dengliang@cqmu.edu.cn](mailto:dengliang@cqmu.edu.cn) Tel.: +86-023-8901-2964 Fax: +86-023-8901-2964

**Supporting figure legends**

**Figure S1. Runx2 was upregulated in human liver fibrosis**

(A) The mRNA expression of Runx2 in obese, steatosis, or NASH human liver tissues compared to control samples in GSE48452 cohorts. (B) The mRNA expression of Runx2 in human liver tissues with different fibrosis stages (FS) in GSE33258 cohorts. **P* <0.05, ***P* <0.01, ****P* <0.001, ns, non-significant.

**Figure S2. Runx2 was progressively increased during the mice liver fibrosis progression.**

(A) Representative photomicrographs of Masson and IHC staining of α-SMA and Runx2 in liver tissues from mice treated with CCl_4_ for 1 weeks and 6 weeks. Scale bars, 100μm. (B) Quantification of positive staining areas was measured by Image J software. (C) qRT-PCR analysis of mRNA level of collagen I, α-SMA, and Runx2. Data are mean ± SEM. n =3 per group. **P* < 0.05 versus controls. *^#^P*<0.01.

**Figure S3. Identification of HSCs and MFBs by scRNA-seq.**

Non-parenchymal cells (NPCs) from mice livers were extracted and cultured for 24 hours or 120 hours *in vitro* for scRNA-seq, and the marker genes for each cell type by performing differential gene expression and analysis of 13 populations and 5 distinct cell lineages were subsequently identified. Next, the endothelial cells, Kupffer cells, and leukocytes were excluded from our dataset to identify clusters of resting HSCs and myofibroblasts. (A) UMAP embedding of mouse liver NPCs after a specific time of culture in vitro. Cells are colored by culture time and cell origin. Regulon activity-based UMAP colored by the regulon activity of Runx2 showing the cell type specificity of regulons. (B) The expression and distribution of Runx2, α-SMA, Col1a1, and Col3a1 in myofibroblasts in UMAP plots. (C) Differentially expressed genes in HSCs compared to myofibroblasts. Log fold change (avg-logFC) gene expression of the top 20 marker genes for each cluster.

**Figure S4. Runx2 deficiency inhibited CCl_4_-induced liver fibrosis**

A. Representative images of Masson staining in liver tissues from the mice treated with CCl_4_ for 4 weeks after injected by Lenti-ctrl or Lenti-shRunx2 (100μl per mouse, 1×10^12^ V.g/mL, tail vein). Quantification of positive staining areas was measured by Image J software. Scale bars, 100μm. B. qRT-PCR analysis of mRNA level of α-SMA. Data are mean ± SEM. n =3 per group. **P* < 0.05 versus to controls.

**Figure S5. Identification of Cre/loxP-mediated recombination of the floxed Runx2 allele.**

(A) *Runx2^f/f^* female mouse crossed with *Runx2^f/+^ PDGFRβ Cre^+/-^* male mice to generate Runx2 ablation littermates, but all of the *Runx2^f/f^ PDGFRβ Cre^+/-^* littermates (red frame) were postnatal dead. (B) *Runx2^f/f^* female mouse crossed with *Runx2^f/+^ GFAP Cre^+/-^* male mice to generate Runx2 ablation littermates, most of the littermates were alive and *Runx2^f/f^ GFAP Cre^+/-^* (red frame) littermates were used for *in vitro* experiments.

**Figure S6. HSCs-specific ablation of Runx2 alleviates DDC-induced or MCD-induced liver fibrosis.**

*Runx2^f/+^* and *Runx2****^△/+HSCs^*** mice were fed with 0.1% DDC for 4 weeks to induce cholestasis, while fed with MCD for 8 weeks to induce NASH. (A) Western-blot assay showed the protein levels of α-SMA and Collagen I in liver tissues from *Runx2^f/+^* and *Runx2****^△/+HSCs^*** mice treated with DDC or MCD. B. qRT-PCR analysis of mRNA levels of Runx2, Collagen I, and α-SMA. Data presented are means ± SEM, n=3 per group. *^*^P*<0.05 versus controls.

**Figure S7. Hepatocytes-specific deletion of Runx2 had no effect on CCl_4_-induced liver fibrosis.**

(A) Schematic showing the strategy for generating hepatocytes (HCs)-specific deletion of Runx2 mice (*Runx2^△/△HCs^*). (B) Representative photomicrographs of Masson staining in liver tissues from the *Runx2^f/f^* or *Runx2****^△/△HCs^*** mice treated with Olive or CCl_4_ for 4 weeks. Scale bars, 100μm. (C) Positive staining areas were measured by Image J software. (D) qRT-PCR analysis of mRNA level of Runx2, Collagen I, α-SMA and TGF-β1. Data are mean ± SEM. n =3 per group.

**Figure S8. Runx2 was successfully overexpressed in the liver.**

Mice were injected with HBAAV-ctrl or HBAAV-Runx2 (1×10^12 vg/mL, portal vein) for 3 weeks. (A) The transfection efficiency was determined by fluorescence observation of frozen sections of mice after sacrifice. Scale bars, 50μm. (B) Primary HSCs and HCs were isolated and the mRNA expression of Runx2 was detected by qRT-PCR analysis. Data are mean ± SEM. n =3 in group. **P* < 0.05 versus controls.

**Figure S9. Runx2 overexpression triggered quiescent HSCs proliferation in mice.**

IHC staining of GFAP (quiescent HSCs marker) in the mouse livers. Quantification of positive staining areas were measured by Image J software. Scale bars, 50μm. n =3. **P* < 0.05 versus controls.

**Figure S10. HSCs-specific overexpression of *Runx2* exacerbates CCl_4_-induced liver fibrosis.**

(A,B)Protein and mRNA analysis of Runx2 in primary HSCs, KCs, LSECs, and HCs isolated from the mice after being injected by VA-Lip-Runx2 or VA-Lip-Ctrl(0.75 mg/kg) for 1 weeks. Data are mean ± SEM. n =3. **P* < 0.05 versus controls. (C, D)The mRNA and protein expression of Runx2, α-SMA and Collagen I were measured by qRT-PCR and western-blot assay in VA-Lip-Runx2 or VA-Lip-Ctrl mice treated by CCl_4_ for 4 weeks. Data are means ± SEM. n=3. **P*<0.05 versus controls. (E,F)Representative images of Masson and IHC staining of Collagen I and α-SMA in VA-Lip-Runx2 or VA-Lip-Control mice treated with CCl_4_ for 4 weeks. Quantification of positive staining areas was measured by Image J software. Scale bars, 100μm. (n=3) Data are mean ± SEM. **P* < 0.05 versus controls.

**Figure S11. Runx2 deletion dramatically alleviated CCl_4_-induced liver fibrosis**

(A) Representative photomicrographs of Masson staining in liver tissues from the mice treated with CCl_4_ for 4 weeks in *Runx2^f/f^* or *Runx2^△/△HSCs^* mice. Positive staining areas were measured by Image J software. Scale bars, 200μm. (B) qRT-PCR analysis of mRNA level of α-SMA. Data are mean ± SEM. n =3. **P* < 0.05 versus controls.

**Figure S12. Runx2 regulated HSCs activation and proliferation *in vitro*.**

Mouse primary HSCs, human HSCs cell line (LX2), and mouse HSCs cell line (mHSCs) were utilized for experiments. siRNA of Runx2 was transfected into cells to reduce the expression of Runx2, while pcDNA3.1-Runx2 was used to overexpress Runx2 in cells. siRNA of scramble and pcDNA3.1-ctrl were utilized as control respectively. (A, B). The mRNA expression of Runx2 and α-SMA were detected by qRT-PCR analysis. (C) The percentage of exponentially growing in each cell cycle phase of mHSCs was measured by Flow cytometry analysis. (D) Cell Counting kit-8 analysis was performed to test the proliferation of mHSCs. Data are means ± SEM. n=3. *^*^P*<0.05 versus controls.

**Figure S13. Runx2 influenced abundant genes and signaling pathways in HSCs.**

(A) The distribution of DNA binding domain of Runx2 in HSCs. (B) Functional enrichment analysis of Runx2 ChIP-seq peaks.

**Figure S14. Circos plot displaying correlations between *Runx2, Itgav, TGF-β1, ACTA2 （α-SMA）,Col1a1* and *PKA* expression levels based on their TPM values.**

Red links represent positive correlations, blue links represent negative correlations, and white indicates no correlation. The width and color intensity of the links correspond to the strength of the correlation between the respective genes.

**Figure S15. Itgav knockdown inhibited the activation of HSCs caused by Runx2 overexpression *in vitro*.**

Primary HSCs were isolated from the *HBAAV-ctrl* or *HBAAV-Runx2* mice and transfected with scramble or siItgav *in vitro*. (A, B) The mRNA and protein level of Itgav and α-SMA was detected by qRT-PCR and Western-blot assay. Data are means ± SEM. n=3. *^*^P*<0.05 versus controls. *^#^P*<0.05.

**Figure S16.HSC-specific knock-in of *siItgav* blocks the aggravation of CCl_4_- induced liver fibrosis caused by Runx2 overexpression.**

(A, B)Protein and mRNA analysis of Itgav in primary HSCs, KCs, LSECs, and HCs isolated from the mice after being injected by VA-Lip-siItgav or VA-Lip-Ctrl (0.75 mg/kg) for 1 weeks. Data are mean ± SEM. n =3. **P* < 0.05 versus controls. (C, D)The mRNA and protein expression of α-SMA was measured by qRT-PCR and western-blot assay in VA-Lip-Ctrl+VA-Lip-Ctrl, VA-Lip-Runx2+VA-Lip-Ctrl, VA-Lip-Ctrl+VA-Lip-*siItgav* or VA-Lip-Runx2+VA-Lip-*siItgav* mice treated by CCl_4_ for 4 weeks. Data are means ± SEM. n=3. **P*<0.05 versus controls. (E, F)Representative images of Masson and IHC staining of Collagen I and α-SMA in VA-Lip-Runx2+VA-Lip-*siItgav* or VA-Lip-Runx2+VA-Lip-Ctrl mice treated with CCl_4_. Quantification of positive staining areas was measured by Image J software. Scale bars, 100μm. (n=3) Data are mean ± SEM. **P* < 0.05 versus controls, *^#^P*<0.05.

**Supplementary Materials and Methods**

**Animal models**

C57BL/6J mice were fed a standard diet (control, Trophic Animal Feed High-Tech Co Ltd, China) or high-fat diet (HFD, Trophic Animal Feed High-Tech Co Ltd, China) for 4 months or 12 months to induce NAFLD or NASH related liver fibrosis.^(1)^ C57BL/6J mice or transgenic mice were injected with Carbon tetrachloride (CCl_4_, 5 μl/g body weight) solution in olive oil twice a week for 2 weeks, 4 weeks or 8 weeks to induce chronic liver injury or fibrosis by intraperitoneal injection (IP). Transgenic mice were fed with 0.1% DDC (Trophic Animal Feed High-Tech Co Ltd, China) for 4 weeks to induce cholestasis-related liver fibrosis, and fed with MCD (Trophic Animal Feed High-Tech Co Ltd, China) for 8 weeks to induce NASH related liver fibrosis.

**CWHM-12 treatment for fibrotic mice**

CWHM-12 (APExBIO TECHNOLOGY, USA) or vehicle (50% DMSO) were delivered by implantable ALZET osmotic minipumps (Durect, Cupertino, USA). *HBAAV-control* mice and *HBAAV-Runx2* mice were given CCl_4_ I.P. twice weekly for 2 weeks, then Alzet minipumps containing either CWHM-12 (100mg/kg/day) or vehicle were inserted, followed by a further 2 weeks of CCl_4_ I.P. twice weekly.

**Histopathologic analysis and Immunohistochemical analysis**

The liver tissues of mice or humans were fixed in 4% paraformaldehyde and cut into thick sections (4-μm) after embedded in the paraffin. Tissue sections were stained with hematoxylin and eosin (H&E) and Masson’s Trichrome (Masson) for routine histological examination. For immunohistochemistry staining, post dewaxing in xylene and dehydrating in alcohol, sections were separately stained with antibodies Runx2, α-SMA, GFAP, or Collagen I. For immunofluorescence staining, primary HSCs fixed with 4% paraformaldehyde or liver tissues sections were co-stained with indicated antibodies of Runx2, α-SMA, CD31, or F4/80 with detection by secondary antibodies (ThermoFisher, USA) labeled with either Alexa 555 or Alexa 488 according to the manufacturer’s instructions. The nuclei were stained with DAPI. Percentages of positive areas for the Masson staining or immunostaining of the total image area were measured by Image J software. The sources and dilutions of antibodies are provided in Table S4.

**Cell lines culture**

The human HSCs line LX-2 and mouse HSCs line mHSCs were purchased from the Cell Bank of Chinese Academy of Sciences and cultured in Dulbecco’s modified Eagle’s medium (DMEM, Gibco, USA) with 10% fetal bovine serum (FBS, Gibco, USA). The cells were maintained at 37°C in a humidified 5% CO_2_ atmosphere.

**Isolation and culture of primary mice HSCs, HCs, KCs and LSECs**

The procedure of extracting primary mouse hepatic stellate cells (HSCs), hepatocytes (HCs), Kupffer cells (KCs) and liver sinusoidal endothelial cells (LSECs) was performed according to our previous experience ^(1,2)^ and other reports^(3-8)^ with a few modifications. C57BL/6 male mice aged 6 weeks (n=3-5) were used for primary cell isolation. After mice was anesthetized and laparotomized, collagenase Ⅳ (0.5mg/ml, Solarbio, China) was infused into the mouse liver via the inferior vena cava until the liver became soft. For HCs and HSCs, the liver was then separated into a culture dish containing the mixture of collagenase Ⅳ and DNAse I solution (20ug/ml, Solarbio, China) for 10 min digestion and then filtrated through a 200‐μm pore size filter to remove undigested tissue. The cell suspension was collected into a 50ml sterile centrifuge tube and centrifuged at 4℃ 50g for 3 mins. HCs were mainly in the substrate while the HSCs were in supernatant because of its light weight. HCs were purified in 48% percoll (Biosharp, China) with centrifugation at 50g for 10 minutes and washed once with PBS. Then, HCs were seeded in culture dishes with William’s E Medium (Gibco, USA) containing 5% FBS (Hyclone, USA), 1% penicillin-streptomycin, 2 mM L-glutamine, 100 nM dexamethasone and 100 nM insulin. HSCs in supernatant were centrifuged at 4°C at 500g for 7 minutes to pellet HSCs. HSCs were suspended in 12.5 ml DMEM (Gibco, USA) and mixed softly with7.5ml Optiprep(Serumwerk Bernburg AG, Germany). Then, Hank’s solution (Gibco,USA) was added to the upper layer slowly without mixing. The sample was then centrifuged at 1400g for 20 mins at 4 °C and washed with PBS once. The purified HSCs were seeded in cell culture dishes with DMEM containing 3% FBS (Hyclone, USA). For KCs and LSECs, the preparation of hepatic cell suspension was the same as HCs and HSCs. The suspension was centrifuged at 4°C 500g for 7 minutes, suspended in 6 ml RPMI-1640 medium (Gibco, USA), then the suspension was transferred onto a two-layer (25/50%) Percoll gradient and centrifuged at 4℃1500g for 20 mins without pausing. LSECs were enriched in the interface between the 25% and the 50% Percoll layer while KCs were in the 50% Percoll layer. These two cells were isolated carefully and washed with PBS, respectively. Then, KCs and LSECs were cultured with RPMI1640 medium containing 10% FBS (Hyclone, USA).

To test the effect of growth factors on Runx2, after 24 hours of isolation, primary HSCs were treated with recombinant mouse TGF-β1 (TG1-M5218, ACROBiosystems Group, China; 5 ng/ml), PDGF-BB (PDB-H4112, ACROBiosystems Group, China; 5 ng/ml) or EGF (EGF-M5265, ACROBiosystems Group, China; 5 ng/ml) for 2 hours followed by DMSO or PKA inhibitor (PKI-6-22, MedChemExpress, USA, 10 nM/mL) for 12 hours, and then the cells were harvested. In the study of the mechanism of Runx2 nuclear translocation, primary HSCs were stimulated with TGF-β1 (5 ng/ml) following the treatment of PKA activator (8-Bromo-cAMP, MedChemExpress, USA; 0.5 nM/mL) or inhibitor for 12 hours, then the cells were harvested for immunoblotting and immunohistochemical analysis.

**Western-blot analysis**

Cells and tissues were lysed using RIPA lysis buffer (ThermoFisher, USA) and applied to 10% SDS-PAGE gels, and blotted onto 0.22 μm PVDF membranes (IPVH00010, Millipore, USA). After blocked with 5% milk, membranes were incubated with indicated antibodies overnight at 4°C. The following primary antibodies were used for the immunoblotting: Runx2, α-SMA, Collagen I, TGF-β1, Itgav, FAK, pFAK, PI3K, pPI3K, and GAPDH. The blots were developed with the HRP-conjugated secondary antibodies and detected with enhanced chemiluminescence reagents (ThermoFisher, USA). The sources and dilutions of antibodies are provided in Table S4.

**Quantitative real-time PCR**

Total RNA extracted from the liver or cells by using Trizol reagent (Qiagen, CA) and reverse-transcribed into complementary DNA (RR037A, TaKaRa, Japan). The polymerase chain reaction (PCR) conditions were performed as previously described.^(1)^ The mRNA expression level of target genes was normalized with the housekeeping gene GAPDH by using 2^-ΔΔCt^ method. The sequences of primers for real-time PCR are shown in Table S5.

**RNA sequencing and analysis**

The total RNA from primary HSCs was extracted by using TRIzol reagent (Invitrogen, Carlsbad, CA). 200 ng of RNA from each sample was used for cDNA library construction as previously reported.^(9)^ The average insert size for the final cDNA library was 300±50 bp, and paired-end sequencing (PE150) on an illumina Novaseq™ 6000 (LC-Bio Technology CO., Ltd., Hangzhou, China) was performed. For the RNA-seq data analysis, the clean reads were extracted from the raw reads with HISAT2, and were compared to the reference genome to generate the mapped reads.^(10)^ Then, the gene expression was analyzed by calculating FPKM. Finally, the volcano plots, gene ontology (GO) analysis and Kyoto Encyclopedia of Genes and Genomes (KEGG) were performed through using DAVID software.

**Single-cell RNA sequencing (scRNA-seq) and analysis**

After dissociation of 8 weeks old C57BL/6J mice livers, non-parenchymal cells (NPCs) were isolated with 11.5% Optiprep as previously described. After culturing for 24 hours or 120 hours *in vitro*, cells were analyzed using the Chromium Single Cell 50 kit (10×Genomics, Pleasanton, CA, USA) following the manufacturer’s protocol. The bioinformatics analysis was performed by OE Biotech Co., Ltd. (Shanghai, China). In brief,^(11)^ the Cell Ranger software pipeline (version 5.0.0) was used to demultiplex cellular barcodes, map reads to the genome and transcriptome using the STAR aligner, down-sample reads normalized aggregate data across samples, and produce a matrix of gene counts versus cells. We processed the unique molecular identifier count matrix with the R package Seurat (version 4.0.0). For a given cluster, FindAllMarkers identified positive markers compared with all other cells. Then, the R package SingleR, a computational method for unbiased cell type recognition of scRNA-seq, with the reference transcriptomic datasets were used to infer the cell of origin of each of the single cells independently and identify cell types.

**Chromatin Immuneprecipitation followed by sequencing (ChIP-seq) and analysis**

Primary HSCs were isolated from *HBAAV-Runx2* mice and cultured for 4 days, then cross-linked with 1% formaldehyde, followed by incubation with 125 mM glycine. The cells were lysed in lysis buffer (0.1% SDS, 1% Triton X-100, 0.15 M NaCl, 1 mM EDTA, and 20 mM Tris (pH 8.0). Next, the lysed cells were sonicated to get an average size of 200-500 bp chromatin fragments. Chromatin was immunoprecipitated with antibody against Runx2 (ab236639, Abcam, UK). DNA was eluted and the cross-links were reverted. Finally, DNA was purified by the QIAquick PCR Purification Kit (QIAGEN, Germany) and quantified by qRT-PCR. ChIP-seq libraries were constructed with NEBNext Ultra II for DNA and sequenced to 150 bp paired-end with Illumina HiSeq machine.^(12)^

**Lenti-virus, plasmid, siRNA construction, and transfection**

The short hairpin RNA (shRNA) lentiviruses targeting the mice Runx2 gene (GenBank accession no. NM_001146038.2) was designed and constructed by Genechem (China) as previously described,^(1)^ Lenti-ctrl or Lenti-shRunx2 (100μl per mouse, 1×10^12^ V.g/mL, tail vein) were injected into mice treated with CCl_4_ for 4 weeks. The plasmid pCDNA 3.1-Runx2 and pGL3-Itgav were constructed by Knorigene Technologies Company (China). In brief, the full-length fragment regions of mouse Runx2 were cloned into the pCDNA3.1 vector. And the Itgav promoter fragment regions of mouse Itgav were cloned into the pGL3-Basic vector. Short interfering RNA (siRNA) vectors targeting mouse Runx2, human Runx2, and mouse Itgav were designed and constructed by Genepharma (Shanghai, China). Lipofectamine 2000 (ThermoFisher, USA) was used as the transfection reagent according to the manufacturer’s protocols. The target sequences are shown in Table S3. VA-Lip-Runx2, VA-Lip-*siItgav* and VA-Lip-control were prepared according to previous reports^(13)^. Briefly, VA solution was [comprise](javascript:;)d of 5 mg VA (Sigma-Aldrich, USA) and 50 μl DMSO. Then 0.14 μmol lipotrust solution (Hokkaido System Science, Japan) was added to 280 nmol VA solution and mixed by vortexing at 25°C. 12.24 nmol control-vector, siItgav or Runx2-plasmid was added into VA-Lip solution with stirring at 25°C separately. The VA-Lip solution was filtered. Fractions were collected and the material trapped in the filter (ThermoFisher, USA) was reconstituted with PBS to achieve the desired dose for in vivo use. Mice were intravenouslyinjected with VA-Lip-Runx2, VA-Lip-*siItgav* or VA-Lip-control combined with CCl_4_ to induce liver fibrosis. VA-Lip-Runx2, VA-Lip-*si**Itgav* or VA-Lip-control (0.75 mg/kg) were administered 2 times a week for 4 weeks after CCl_4_ injection for the first time. All experimental procedures were approved by the institutional and local committee on the care and use of animals of Chongqing Medical University and all animals received humane care according to the National Institutes of Health (USA) guidelines.

**Luciferase assay**

The Itgav promoter luciferase reporter assays were carried out in mHSC. Briefly, cells were transiently transfected with pCDNA 3.1-Runx2 or pCDNA 3.1-ctrl to overexpress Runx2 and cotransfected with pGL3-Itgav promoter or pGL3-ctrl. After transfection for 24 hours, the cells were lysed for luciferase activity with a dual luciferase assay kit (E1910; Promega, USA) according to the manufacturer’s instructions as described. Luciferase signals were calculated relative to the pRL-TK-renilla-luciferase.

**Cell Cycle and Proliferation Assay**

For Cell Cycle assay, primary HSCs were isolated from *Runx2^f/f^*  mice, *Runx2^△/△HSCs^* mice, *HBAAV-ctrl* mice, or *HBAAV-Runx2* mice separately, and mHSCs were transfected with siRNA of scramble or Runx2 and cultured for 48 hours. Next, the cell cycle of the cells was examined by using PI staining kit (C1052, Beyotime Biotechnology, China) on a BD FACSCalibur^TM^ flow cytometer following the manufacturer’s instructions. For cell proliferation assay, 4×10^3^ mHSCs were seeded in 96-well microplates and transfected with pCDNA 3.1-ctrl or pCDNA 3.1-Runx2. After 1 day, 3 days, 5 days, and 7 days culturing, the cell viability was measured by the Cell Counting Assay Kit-8 (C0037, Beyotime Biotechnology, China) according to the manufacturer’s instructions.

**Data collection and preprocessing**

The GEO database (<http://www.ncbi.nlm.nih.gov/geo>) was used to obtain the gene expression profiles. The gene expression profiles of GSE25097, GSE103580, GSE49541, GSE48452, GSE33258 and GSE3143 were downloaded. Pre-processing programs (including background adjustment, normalization, summarization, and gene chip probe annotation) were executed using R language (Version 3.6.3; https://www.R-project.org). For multiple probes corresponding to a gene, the average expression value was taken as the gene expression value.

**DATA CORRELATION**

In this study, we utilized the public RNA-seq dataset GSE154055(liver fibrosis) to analyze gene expression patterns and investigate the relationships between different genes. Raw sequence reads were obtained from the Gene Expression Omnibus (GEO) database and processed using the Nextflow RNA-seq pipeline. The pipeline facilitated quality control, read alignment, and quantification of gene expression levels in terms of Transcripts Per Million (TPM) values.The processed data consisted of gene expression TPM values for each sample in the dataset. To study the correlation between genes, we calculated the pairwise Pearson correlation coefficients for the selected genes using their TPM values. The correlation matrix was visualized using a circos plot generated with the circlize library in R.

The correlation matrix displays the pairwise correlations between the genes *Runx2, Itgav, TGF-β1, ACTA2,* *Col1a1, and PKA*. The values in the matrix range from -1 to 1, where 1 represents a perfect positive correlation, 0 represents no correlation, and -1 represents a perfect negative correlation.The matrix shows that Runx2 is highly correlated with *Itgav* (r=0.98), *TGF-β1*(r=0.91), and *Col1a1*(r=0.97). *ITGAV* is also highly correlated with *TGF-β1*(r=0.87) and *Col1a1*(r=0.94). These results suggest that there may be some functional relationships between these genes, and that they may be co-regulated or involved in similar biological pathways.In contrast, *ACTA2* is only weakly correlated with the other genes, with the highest correlation coefficient being 0.71 with *Itgav. PKA* is also weakly correlated with the other genes, with the highest correlation coefficient being 0.89 with *COL1A1*. These results suggest that *ACTA2* and *PKA* may have different functions or may be regulated differently than the other genes in this set.

**Statistics**

SPSS version 19.0 was employed to analyze the obtained data. Quantitative data has been indicated as the mean ± SEM of at least three independent experiments of *in vivo* studies and each cell experimental group. For two-group comparisons, statistical significance was calculated using Student's t-tests for normally distributed variables and the Wilcoxon rank-sum test for nonnormally distributed data. Kruskal-Wallis tests and one-way analysis of variance were employed as nonparametric and parametric procedures for comparisons of more than two groups, respectively. The difference of *P* < 0.05 was regarded as statistically considerable.

**References:**

1. Zhong L, Huang L, Xue Q, Liu C, Xu K, Shen W, Deng L. Cell-specific elevation of Runx2 promotes hepatic infiltration of macrophages by upregulating MCP-1 in high-fat diet-induced mice NAFLD. J Cell Biochem. 2019 Jul;120(7):11761-11774.

2. Zhao J, Bai J, Peng F, Qiu C, Li Y, Zhong L. USP9X-mediated NRP1 deubiquitination promotes liver fibrosis by activating hepatic stellate cells. Cell Death Dis. 2023 Jan 19;14(1):40. .

3. MEDERACKE I, DAPITO D H, AFFO S, et al. High-yield and high-purity isolation of hepatic stellate cells from normal and fibrotic mouse livers [J]. Nat Protoc, 2015, 10(2): 305-15.

4. LI P Z, LI J Z, LI M, et al. An efficient method to isolate and culture mouse Kupffer cells [J].

Immunol Lett, 2014, 158(1-2): 52-6.

5. GUO Q, FURUTA K, ALY A, et al. Isolation and Characterization of Mouse Primary Liver

Sinusoidal Endothelial Cells [J]. J Vis Exp, 2021, (178).

6. BARTNECK M, TOPUZ F, TAG C G, et al. Molecular response of liver sinusoidal endothelial cells on hydrogels [J]. Mater Sci Eng C Mater Biol Appl, 2015, 51: 64-72.

7. CHARNI-NATAN M, GOLDSTEIN I. Protocol for Primary Mouse Hepatocyte Isolation [J]. STAR

Protoc, 2020, 1(2): 100086.

8. Zhang Q, Qu Y, Li Z, Zhang Q, Xu M, Cai X, Li F, Lu L. Isolation and Culture of Single Cell Types from Rat Liver. Cells Tissues Organs. 2016;201(4):253-67.

9. Mortazavi A, Williams BA, McCue K, Schaeffer L, Wold B. Mapping and quantifying mammalian transcriptomes by RNA-Seq. Nat Methods 2008;5:621-628.

10. Kim D, Langmead B, Salzberg SL. HISAT: a fast spliced aligner with low memory requirements. Nat Methods 2015;12:357-360.

11. Butler A, Hoffman P, Smibert P, Papalexi E, Satija R. Integrating single-cell transcriptomic data across different conditions, technologies, and species. Nat Biotechnol 2018;36:411-420.

12. Hansel-Hertsch R, Spiegel J, Marsico G, Tannahill D, Balasubramanian S. Genome-wide mapping of endogenous G-quadruplex DNA structures by chromatin immunoprecipitation and high-throughput sequencing. Nat Protoc 2018;13:551-564.

13. Sato Y, Murase K, Kato J, Kobune M, Sato T, Kawano Y, Takimoto R, Takada K, Miyanishi K, Matsunaga T, Takayama T, Niitsu Y. Resolution of liver cirrhosis using vitamin A-coupled liposomes to deliver siRNA against a collagen-specific chaperone. Nat Biotechnol. 2008 Apr;26(4):431-42.
